# A Microphysiological Interface of Skeletal Myobundles and Inflamed Adipose Tissue for Recapitulating Muscle Dysfunction in an Obese Microenvironment

**DOI:** 10.1002/adhm.202502711

**Published:** 2025-11-10

**Authors:** Seunggyu Kim, Tianxin Cao, Zhengpeng Wan, Jaesang Kim, Zhuxuan Li, Legairre A. Radden II, Rakesh Santhanam, Eunkyung Clare Ko, Tatsuya Osaki, Sarah Spitz, Hyunmin Moon, Maria Proestaki, Seokbeom Roh, Gyudo Lee, Jessie S. Jeon, Curtis R. Warren, Roger D. Kamm

**Affiliations:** ^1^ Department of Mechanical Engineering Massachusetts Institute of Technology Cambridge Massachusetts 02139 USA; ^2^ Department of Digital Healthcare Engineering Korea University Sejong 30019 Republic of Korea; ^3^ Cardiovascular‐Renal Metabolic Diseases Research Department Boehringer Ingelheim Pharmaceuticals, Inc Ridgefield Connecticut 06877 USA; ^4^ Department of Biological Engineering Massachusetts Institute of Technology Cambridge Massachusetts 02139 USA; ^5^ Department of Mechanical Engineering Korea Advanced Institute of Science and Technology Daejeon 34141 Republic of Korea; ^6^ Oncology Research Department Boehringer Ingelheim Pharmaceuticals, Inc Ridgefield Connecticut 06877 USA; ^7^ Immunology and Respiratory Research Department Boehringer Ingelheim Pharmaceuticals, Inc Ridgefield Connecticut 06877 USA; ^8^ Computational Innovation Department Boehringer Ingelheim Pharma GmbH & Co. 88400 Biberach an der Riss Germany; ^9^ Picower Institute for Learning and Memory Massachusetts Institute of Technology Cambridge Massachusetts 02139 USA; ^10^ Department of Biotechnology and Bioinformatics Korea University Sejong 30019 Republic of Korea; ^11^ Digital Healthcare Center Sejong Institute of Business and Technology Korea University Sejong 30019 Republic of Korea

**Keywords:** 3D engineered muscle tissue, adipose‐muscle crosstalk, inflammatory obese microenvironment, microphysiological systems

## Abstract

Systemic inflammation associated with obesity impairs skeletal muscle function through paracrine signaling from intermuscular adipose tissue—adipose depots situated between adjacent skeletal muscle groups—as well as from visceral adipose tissue, which consist of infiltrating macrophages surrounding inflamed adipocytes. These signals disrupt metabolic homeostasis and reduce muscle contractility, yet existing models are limited in their ability to recapitulate the crosstalk between skeletal muscle and inflamed adipose tissue in a physiologically relevant context. To address this, a human cell‐based microphysiological system is developed that combines engineered muscle tissue (EMT) with an inflamed adipose‐macrophage co‐culture (IAMC) to model obesity‐associated muscle dysfunction. EMTs, derived from human myoblasts on micropillar devices, self‐assembled into 3D contractile myobundles. IAMC are generated by co‐culturing inflamed adipocytes with pro‐inflammatory M1‐polarized macrophages, thereby recapitulating the obese inflammatory microenvironment. EMT‐IAMC co‐culture significantly reduced muscle contractility. Furthermore, cytokine profiling revealed elevated levels of pro‐inflammatory mediators, and transcriptomic analysis showed metabolic reprogramming in EMTs, including upregulation of genes linked to fatty acid transport and insulin resistance. Collectively, these findings underscore the detrimental effects of inflamed adipose tissue on skeletal muscle function and suggest the potential utility of an interfaced platform for studying adipose‐muscle interactions and screening therapies for obesity‐related muscle dysfunction.

## Graphical abstract

A human cell‐based microphysiological system integrates engineered muscle tissues with an inflamed adipose–macrophage niche to model obese microenvironment‐induced muscle dysfunction. Muscle contraction is quantified by pillar deflection coupled with computational stiffness estimation. Secretome and transcriptomic profiling reveal inflammation‐mediated metabolic shifts, highlighting the platform's capability to couple functional readouts with molecular insights in preclinical research.

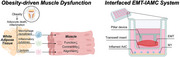

## 1 Introduction

Obesity is a global health crisis associated with chronic low‐grade inflammation and profound metabolic dysfunction. Impaired skeletal muscle functionality is associated with obesity, compounding its effects of obesity on mobility, metabolism, and overall quality of life.^[^ ^1^ ^]^ Skeletal muscle dysfunction arises from both systemic inflammations driven by localized paracrine effects of intermuscular adipose tissue and hypertrophied adipose depots, particularly visceral fat, which harbors the highest density of inflamed adipocytes (AdCs) and macrophages, and secretes pro‐inflammatory cytokines that propagate systemic insulin resistance and muscle atrophy.^[^ ^2^, ^3^ ^]^ The intermuscular adipose tissue, characterized as adipose depots interspersed between and around muscle fibers, plays a unique and active role in obesity‐related muscle dysfunction.^[^ ^4^ ^]^ This depot secretes a range of bioactive factors, including inflammatory cytokines, chemokines, and adipokines such as tumor necrosis factor‐alpha (TNF‐α) and C‐C motif chemokine ligand 2 (CCL2), which disrupt the physiological balance of muscle tissue, leading to insulin resistance, atrophy, and reduced contractility.^[^ ^5^, ^6^ ^]^

The pathological effects of the inflamed adipose depots on skeletal muscle are largely mediated through paracrine signaling pathways.^[^ ^7^, ^8^ ^]^ Adipose tissue‐derived factors, such as interleukin‐6 (IL‐6) and leptin, directly or indirectly interfere with muscle metabolism by inducing pro‐inflammatory signaling cascades and oxidative stress. This paracrine communication fosters a vicious cycle in which muscle fibers become dysmetabolic, including loss of metabolic flexibility, impaired glucose uptake, and mitochondrial dysfunction. Additionally, the infiltration of inflammatory macrophages into adipose tissue exacerbates the inflammatory response and amplifies the secretion of harmful cytokines, further impairing muscle function.^[^ ^9^, ^10^, ^11^ ^]^ Despite the growing recognition of these inter‐tissue interactions, studying the complex dynamics between inflamed adipose tissue and muscle functionality remains an unmet need in that it requires complex, physiologically relevant models that mimic the obese microenvironment.

Microphysiological systems (MPS) using human cells have emerged as transformative tools for modeling complex tissue‐tissue interactions under controlled conditions. These systems have successfully recapitulated various aspects of skeletal muscle functionality^[^ ^12^, ^13^, ^14^, ^15^, ^16^, ^17^ ^]^ and adipose‐immune crosstalk.^[^ ^18^, ^19^ ^]^ For instance, previous MPS studies have focused on modeling neuromuscular junctions to study neurological diseases such as amyotrophic lateral sclerosis^[^ ^20^ ^]^ or vascularization to investigate angiogenesis and fluid exchange in engineered muscle tissue.^[^ ^21^ ^]^ Adipose tissue‐based MPS models have also facilitated the study of obesity by recapitulating pro‐inflammatory interactions within inflamed adipose–macrophage co‐cultures (IAMC), characterized by a microenvironment enriched in IL‐6 and TNF‐α.^[^ ^18^, ^19^ ^]^ However, to our knowledge, no existing MPS has investigated the direct interplay between human skeletal muscle and IAMC. This gap in research limits our understanding of how inflammatory adipose environments influence muscle functionality through paracrine signaling.

To address this gap, we developed an interfaced MPS platform that combines engineered muscle tissue (EMT) with the IAMC model to recapitulate the paracrine signaling interface characteristic of the obese inflammatory microenvironment. EMTs, differentiated from human‐induced pluripotent stem cells (hiPSC)‐derived myoblasts, formed contractile 3D myobundles on a micropillar device, which enabled the measurement of muscle contraction force and velocity in response to electric stimulation (E‐Stim). IAMC were modeled by co‐culturing TNF‐α‐treated adipocytes with pro‐inflammatory (M1) macrophages, which recapitulated aspects of the inflammatory adipose tissue environment observed in obesity. By coupling functional EMT contractility measurements with IAMC‐derived inflammatory signals, this model revealed how adipose‐macrophage crosstalk directly impairs muscle performance, offering a physiologically relevant tool to dissect obesity‐driven contractile deficits.

Our EMT‐IAMC platform provides a physiologically relevant model to study the mechanisms underlying muscle dysfunction in obesity. By quantifying muscle force and velocity in the presence of inflammatory cytokines and adipokines, we show how obesity‐associated inflammation impairs skeletal muscle functionality. This work highlights the potential of advanced organ‐on‐chip systems to unravel complex disease mechanisms and accelerate the development of targeted treatments for obesity‐related complications.

## 2 Results

### 2.1 Construction of On‐Device 3D EMT

**Figure 1:**
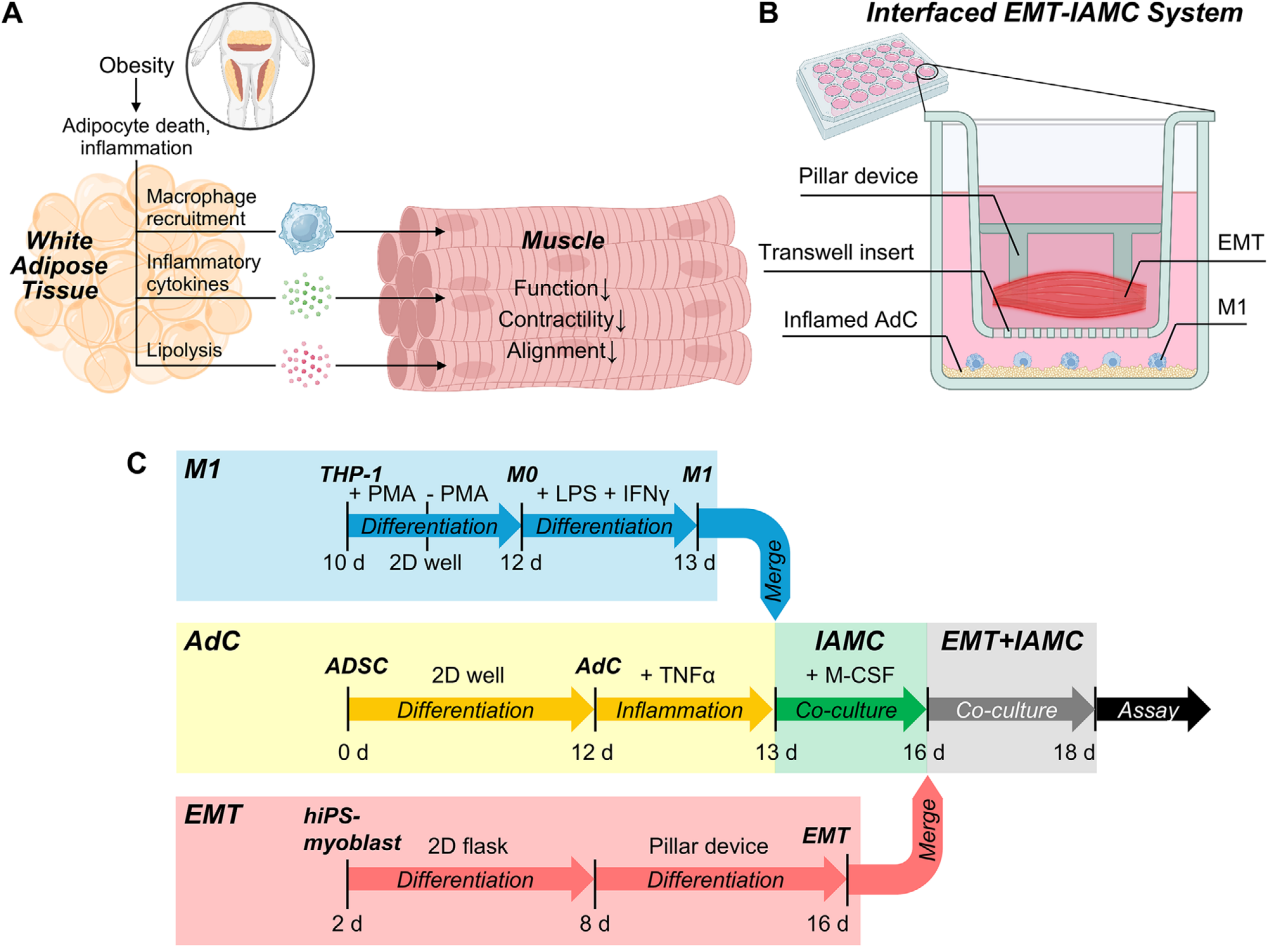
An interfaced system to recapitulate impaired skeletal muscle contractility in an inflamed adipose microenvironment. A) Schematic representation of in vivo muscle in an obese physiological state. B) Schematic of the combined system consisting of EMT‐on‐device and IAMC‐in‐well. C) Experimental timeline for fabricating the EMT‐IAMC co‐culture interface.

In physiology, the functionality of muscle tissue is diminished in an obese microenvironment due to interactions mediated by paracrine signaling pathways (**Figure** **1**A). To investigate the effects of an inflamed adipose tissue microenvironment on skeletal muscle function, we developed a co‐culture system that combines EMT with IAMC, mimicking the pathological features of obesity‐associated dysfunctional muscle tissue (Figure 1B). Briefly, we aimed to fabricate EMTs onto PDMS pillar devices by differentiating hiPSC‐derived myoblasts into 3D myobundles embedded within a collagen‐based hydrogel (Figure 1C). In the meantime, we also aimed to form IAMC by co‐culturing TNF‐α‐treated adipocytes with M1 macrophages, thereby replicating the inflammatory milieu characteristic of obese adipose depots. To establish a paracrine signaling interface between inflamed adipose tissue and skeletal muscle, EMTs cultured on micropillar devices were then transferred to a Transwell insert and co‐cultured above the IAMC‐containing wells. This configuration enabled soluble inflammatory factors secreted by IAMC to diffuse and interact with the muscle tissues without direct physical contact, simulating the paracrine communication observed in vivo. The EMT‐IAMC system thus is able to provide a physiologically relevant model to study obesity‐associated muscle dysfunction and to evaluate the impact of adipose tissue inflammation on skeletal muscle contractility in a controlled, human cell‐based microphysiological environment.

**Figure 2:**
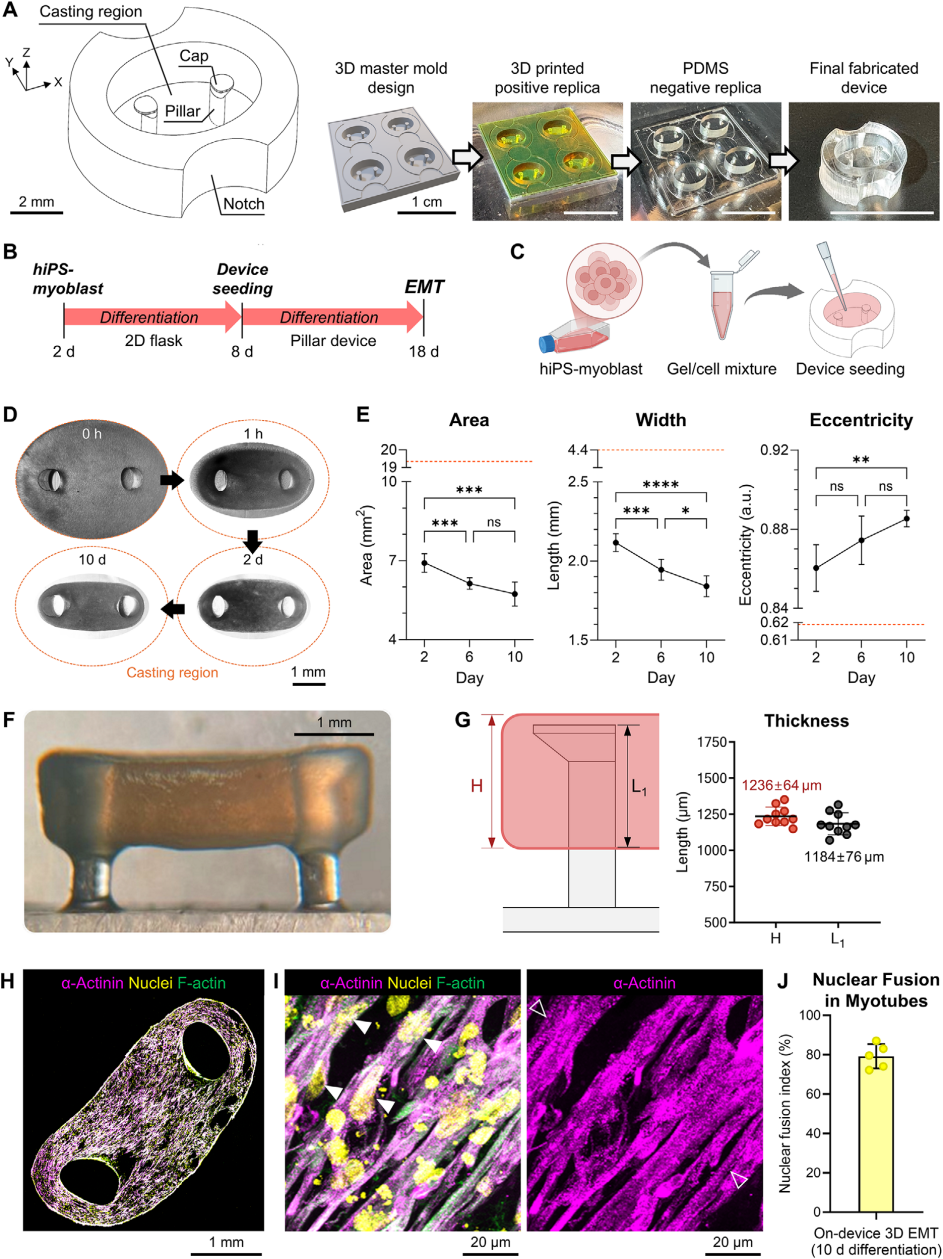
Fabrication of the pillar structure and on‐device reconstruction of 3D EMT. A) Design and fabrication steps of the pillar device. Scale bars 1 cm unless indicated. B) Experimental timeline and C) schematic of 3D EMT fabrication. D) Optical top‐view images of EMT 0 h, 1 h, 2 d and 10 d after gel polymerization, and E) the measurements of area, width, and eccentricity of EMT during on‐device differentiation. The orange dashed lines represent casting region and its corresponding shape measurements. Repeated measures ANOVA followed by Tukey's multiple comparison tests across multiple days. n = 9 independent EMTs. F) Optical side‐view image of EMT at 10 d. G) Schematic representation and measurements of the tissue thickness, H, and anchorage thickness, L_1_, of the EMT. n = 10 independent EMTs. H) Z‐projected immunofluorescence images of a 30 µm‐thick cryosectioned EMT slice stained for sarcomeric α‐actinin, and I) its magnified views. Magenta: sarcomeric α‐actinin; yellow: nuclei; green: F‐actin. Solid and hollow arrows in I) indicate multi‐nucleated myotubes and their sarcomeric Z‐disc striation patterns, respectively. J) Nuclear fusion index in 3D‐cultured myotubes, defined as the ratio of nuclei within multi‐nucleated myotubes to the total number of nuclei. n = 5 independent EMTs.

We designed a Polydimethylsiloxane (PDMS) pillar device comprising two pillars in a gel casting region and notches (**Figure** **2**A). To prevent the EMT from slipping off the pillars due to the tension generated during on‐device differentiation, a wide cap was designed at the top of the pillars (Figure S4, Supporting Information). Additionally, the cross‐section of the pillar was designed to be elliptical to align the pillar deflection with the EMT's long axis. This is supported by the second moments of area of the pillar's cross‐section: 3.6 × 10^−15^ m^4^ versus 9.9 × 10^−15^ m^4^, where the axis of rotation corresponds to the major or minor axis of cross‐section, respectively. The PDMS device was fabricated using a 3D printer and multiple steps of soft lithography, resulting in good consistency between designed and measured pillar dimensions, with a maximum error of 5% (calculated as difference/designed×100) (Figure S5, Supporting Information). HiPSC‐derived myoblasts, embedded in 3D collagen gel within the casting region of the PDMS device (Figure 2B,C), spontaneously compacted the hydrogel around the pillars within 1 h (Figure 2D). The mixture was further compacted into a tightened tissue of smaller area and width, and enhanced elliptical eccentricity over 10 days of differentiation on the device (Figure 2E). This compaction resulted in a hanging 3D EMT anchored to the pillars and their caps (Figure 2F), with an anchorage thickness of 1184 µm (Figure 2G).

The differentiated myotubes within the collagen gel exhibited a multi‐nucleated, elongated morphology aligned along the long axis of the bundle markers of mature myotubes, including anti‐all myosin heavy chain isoforms (MF‐20) and skeletal myosin heavy chain slow isoform (MHC‐slow) (Figure S6, Supporting Information), which are associated with actin filament regulation and muscle contractility. Sarcomeric Z‐disc striation patterns, indicative of mature myotube architecture, were also observed through immunostaining for α‐actinin (Figure 2H,I). Quantitative analysis of our 3D EMT revealed a nuclear fusion index of ≈79.2%, consistent with levels observed in mature myotubes (Figure 2J).^[^ ^20^ ^]^

### 2.2 Estimation of Effective Pillar Stiffness Using Finite Element Analysis

**Figure 3:**
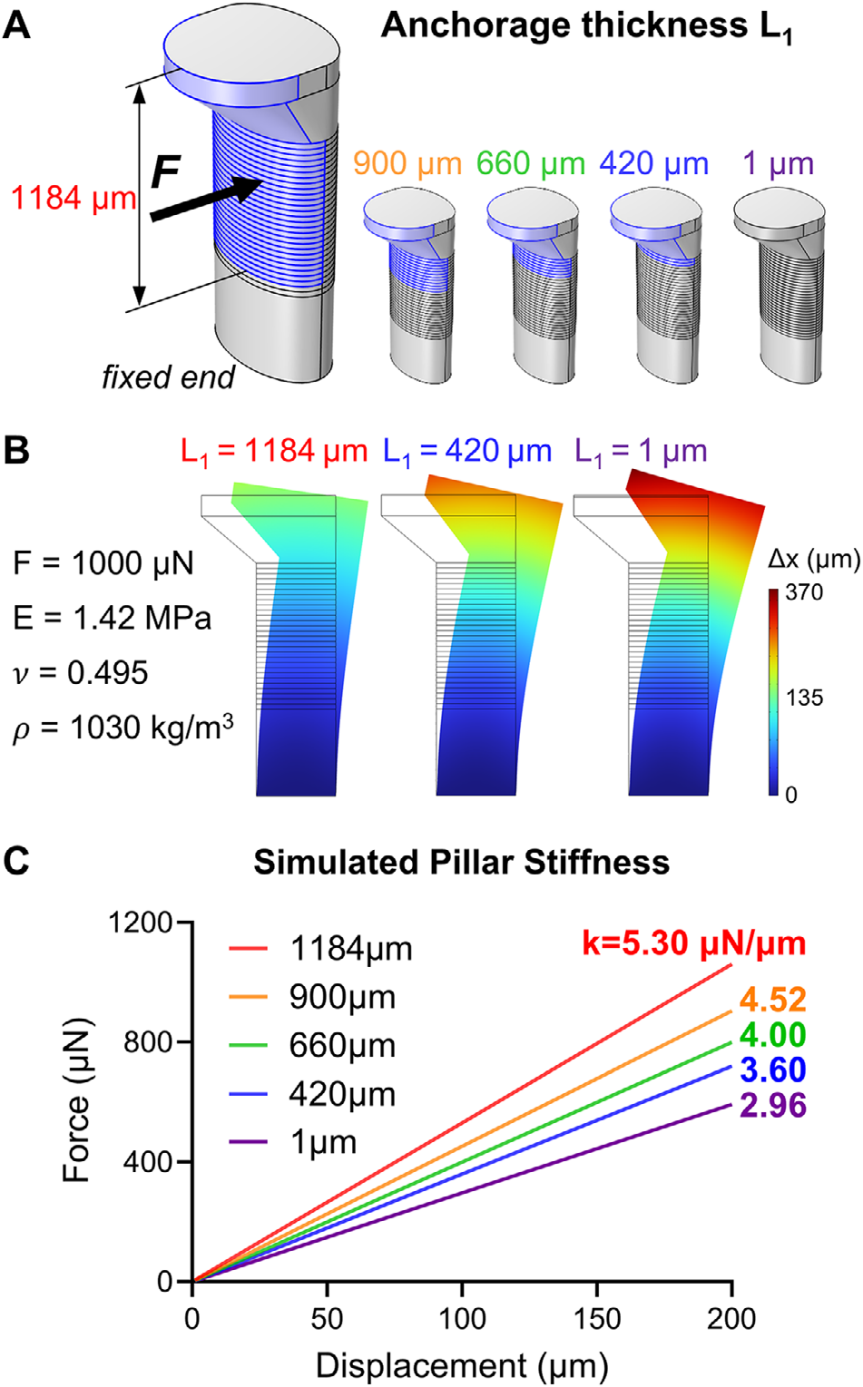
Computational estimation of pillar stiffness as a function of EMT anchorage thickness. A) Schematic representation of EMT‐anchored regions with corresponding thickness, 1184, 900, 660, 420, and 1 µm, denoted as L_1_. B) Simulated pillar deflection under an applied force of 1000 µN, incorporating PDMS material parameters, depending on the anchorage thicknesses of 1184, 420, and 1 µm. C) Simulated force‐displacement curves and the corresponding effective pillar stiffness values as a function of EMT anchorage thickness.

The EMT anchored on the soft PDMS pillar contracted and relaxed repetitively in response to E‐Stim, resulting in the deflection of the pillar. To calculate the magnitude of muscle force generation, we estimated the pillar stiffness by employing finite element analysis (FEA) (**Figure** **3**A). In the computational pillar model, we simulated a linear deflection–force relationship (Figure 3B), using an atomic force microscopy (AFM)‐measured Young's modulus of 1.42 MPa for PDMS (Figure S7, Supporting Information).^[^ ^22^, ^23^ ^]^ Importantly, depending on the anchorage lengths of the muscle band on the pillar, the simulated stiffness ranged from 3.60 to 5.30 µN µm^−1^, corresponding to anchorage thicknesses between 420 and 1184 µm, respectively (Figure 3C). A simulation incorporating a 1 µm anchorage thickness—analogous to the concentrated force applied at the free end of the pillar, as assumed in most previous studies^[^ ^12^, ^24^ ^]^—yielded a stiffness of 2.96 µN µm^−1^, which closely matched the analytical solution of 2.92 µN µm^−1^ (Figures 3C; S8, Supporting Information).

To validate our FEA‐based stiffness estimation, we additionally employed two independent approaches: a hanging‐mass experiment and its corresponding digital twin model (Figure S9, Supporting Information). A 270 µm thread looped beneath the pillar cap suspended calibrated masses (0, 173, 224, and 428 mg) to induce deflection measured by a camera (Figure S9A, Supporting Information), while the digital twin model assumed the thread applied force directly beneath the cap over a contact area equal to its thickness, with all other parameters identical to the previous model (Figure S9B, Supporting Information). The experiment yielded a stiffness of 3.85 ± 0.06 µm µN^−1^, while the model yielded 4.12 µm µN^−1^ (Figure S9C, Supporting Information). The difference between the two results, 6.6% (calculated as difference/model × 100), was likely attributable to experimental variations in thread location and thickness. Given the variability in pillar design and myobundle thickness across studies, our FEA approach appears to be a broadly applicable and reasonable method for estimating stiffness. Accordingly, we used the pillar stiffness of 5.30 µN µm^−1^ (Figure 3C) for all subsequent force measurements, based on the further assumption that the muscle anchorage thickness is constant at 1184 µm (Figure 2G).

### 2.3 Construction of IAMC‐In‐Well

**Figure 4:**
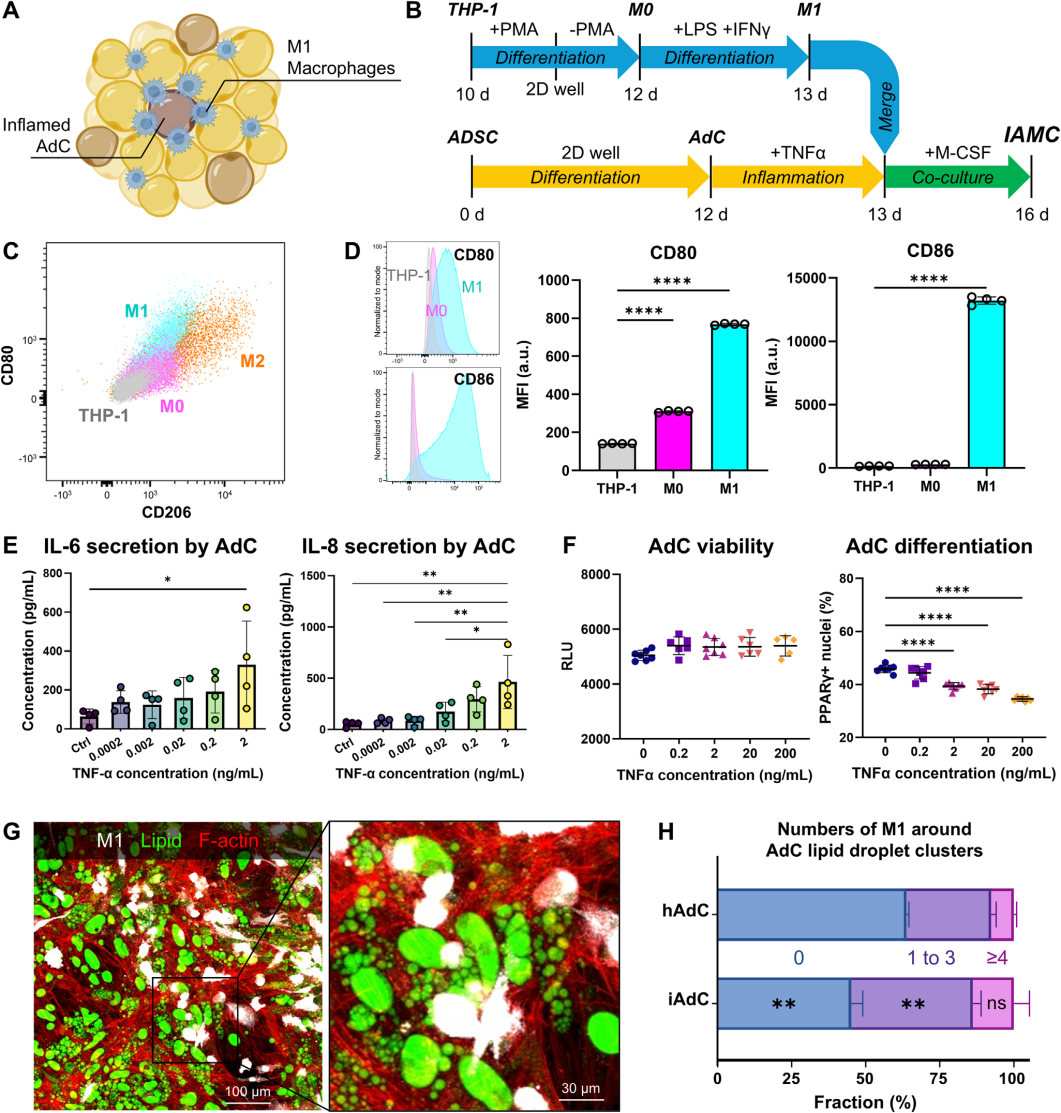
Construction of IAMC‐in‐well in vitro. A) Schematic illustration of IAMC and B) experimental timeline for IAMC construction. C) Flow cytometry double plot of M1‐enriched surface marker CD80 and M2‐specific marker CD206 to distinguish subpopulations of THP‐1 monocyte, M0, M1, and M2 macrophages. D) Surface expression of CD80 and CD86 at different stages of macrophage differentiation (THP‐1 monocyte, M0 and M1 macrophages, respectively). n = 4. E) IL‐6 and IL‐8 secretion from adipocytes after 24 h treatment with multiple concentrations of TNF‐α. One‐way ANOVA followed by Tukey's multiple comparison tests. n = 4. F) Viability and fraction of PPARγ^+^ nuclei of TNF‐α‐treated AdCs across multiple concentrations. One‐way ANOVA followed by Tukey's multiple comparison tests. n = 6‐7. G) Representative image and its magnified views of the IAMC. White: BFP‐labeled M1 macrophages; green: lipid; red: F‐actin. H) Number of M1 macrophages surrounding AdC lipid droplet clusters. hAdC (healthy AdC), iAdC (inflamed AdC). Two‐sample t‐test between respective numbers of M1 groups. n = 3 independent wells.

In an obese microenvironment, M1 macrophages migrate toward inflamed AdCs and form aggregates (**Figure** **4**A).^[^ ^18^, ^19^ ^]^ To mimic the in vivo obese microenvironment, in vitro IAMC were generated by co‐culturing inflamed TNF‐α treated AdCs and M1 macrophages in a multi‐well plate for 3 days (Figure 4B). Initially, we differentiated THP‐1 monocytes into M1 macrophages, verifying the M1 phenotype through the enhanced surface expression of M1 markers CD80 and CD86 (Figures 4C,D; S3, Supporting Information). Concurrently, the absence of M2 macrophages was validated through minimal detection of the M2 marker CD206, which served as a negative control (Figures 4C; S3, Supporting Information). We also differentiated adipose derived stem cells (ADSCs) into AdCs for 12 d and further induced inflammation with TNF‐α. To evaluate dose and duration effects, we performed a TNF‐α dose‐response series with 24 and 48 h treatments. Confirmation of adipokine secretion revealed a TNF‐α concentration‐dependent induction of IL‐6 and IL‐8 (Figure 4E), with no significant differences between 24 and 48 h exposures (Figure S10, Supporting Information). In contrast, other cytokines (CCL2, adiponectin, PAI‐1, and HGF) showed no significant changes across TNF‐α concentrations or treatment durations (Figure S10, Supporting Information). Across the multiple concentrations of TNF‐α, cell viability was maintained while adipogenic differentiation decreased from PPARγ^+^ nuclear staining (Figures 4F; S2, Supporting Information). Based on these results, we selected a concentration of 2 ng mL^−1^ and a treatment duration of 24 h for TNF‐α treatment. Finally, IAMCs were created by directly co‐culturing inflamed AdCs and M1 macrophages for 3 d (Figure 4G). Notably, compared to the healthy AdC group, the inflamed AdC group showed a significantly increased number of M1 macrophages surrounding lipid droplet clusters (Figure 4H). This result may suggest that inflamed AdCs attracted M1 macrophages by secreting chemoattractant cues.

### 2.4 Impaired Muscle Contractility Under IAMC Co‐Culture Interface

**Figure 5:**
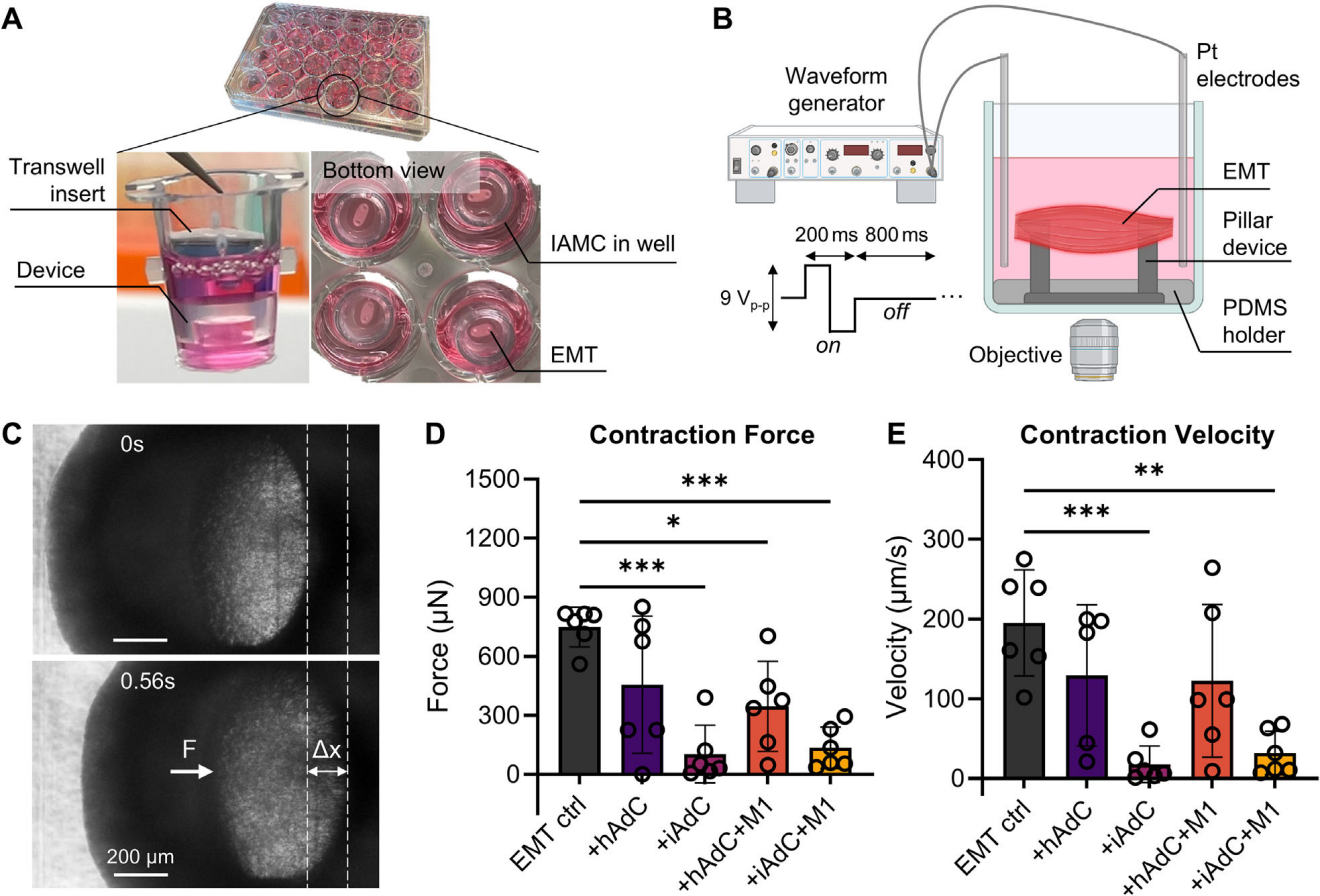
EMT contractility under IAMC co‐culture interface in response to E‐Stim. A) Optical images of the EMT‐IAMC system. B) Schematic of electrical stimulation setup. C) Monitoring of pillar deflection by EMT during E‐Stim. Scale bars 200 µm. D) Contraction force and E) contraction velocity of EMT in response to E‐Stim (1 Hz, 9 V_p‐p_, 20% duty cycle) under various IAMC co‐culture conditions: +hAdC (EMT+healthy AdC), +iAdC (EMT+inflamed AdC), +hAdC+M1 (EMT+hAdC+M1 macrophages), and +iAdC+M1 (EMT+iAdC+M1; IAMC). n = 6 independent EMTs. One‐way ANOVA followed by Tukey's multiple comparison tests.

To evaluate the functional impact of an inflamed adipose tissue microenvironment on skeletal muscle contractility, we established a co‐culture interface between EMT and IAMC (**Figure** **5**A). EMTs, pre‐cultured on micropillar devices for 8 d, were transferred onto Transwell inserts and co‐cultured with IAMC—comprising TNF‐α‐treated AdCs and M1 macrophages—for an additional 2 d. This co‐culture setup was designed to expose EMTs to inflammatory cytokines and metabolites secreted by IAMC, thereby mimicking the pathological paracrine interactions observed in obesity and leading to impaired muscle contractility. After a total of 10 days of differentiation and co‐culture on the device, EMT contractility was assessed in response to E‐Stim. EMTs were stimulated at 1 Hz with a 9 Vp‐p square wave, and the resultant deflection of the micropillars was monitored in real time to quantify contractile force and velocity (Figure 5B,C; Videos S1 and S2, Supporting Information). Muscle contraction force was calculated by multiplying the maximum pillar displacement by the FEA‐derived effective stiffness of the pillar, while contraction velocity was determined by dividing the displacement by the corresponding time interval.

EMTs cultured alone (EMT control) exhibited twitch contractions, generating an average contractile force of 748 ± 101 µN (Figure 5D). In contrast, co‐culture with inflamed adipocytes (+iAdC) or inflamed adipocytes combined with M1 macrophages (+iAdC+M1; IAMC) resulted in substantial reductions in contractile force by ≈86% or 82%, respectively, compared to the control. Co‐culture with healthy adipocytes (+hAdC) showed a milder reduction in force (≈39%), whereas the addition of M1 macrophages to healthy adipocytes (+hAdC+M1) further decreased contractile force by ≈54%.

Consistent with these observations, contraction velocity measurements exhibited similar trends (Figure 5E). Notably, compared to the EMT control group, EMTs co‐cultured with inflamed adipocytes (+iAdC) or IAMC (+iAdC+M1) exhibited significant 91% or 84% decreases in contraction velocity, paralleling the reduction trends observed in contractile force. Co‐culture with healthy adipocytes (+hAdC) or healthy adipocytes combined with M1 macrophages (+hAdC+M1) resulted in nonsignificant and milder reductions in contraction velocity by 34% or 37%, respectively.

Collectively, these findings indicate that the inflammatory signals derived from inflamed adipocytes significantly impair skeletal muscle contractility. The inflamed adipocyte co‐culture (+iAdC) produced reductions in both contractile force and velocity comparable to those observed in the IAMC co‐culture (+iAdC+M1), representing the detrimental effect of an inflamed adipose microenvironment on skeletal muscle function rather than the inflammatory niche created by M1 macrophages. This finding warrants further investigation into the potential amplification loop of inflammation mediated by M1 macrophage signaling and recruitment.

### 2.5 Biochemical Characterization of EMT in IAMC Microenvironment

To investigate the paracrine inflammatory interactions between IAMC and EMT, and their impact on EMT gene expression, we performed biochemical analyses of the co‐culture system by collecting both conditioned media and EMT samples.

**Figure 6:**
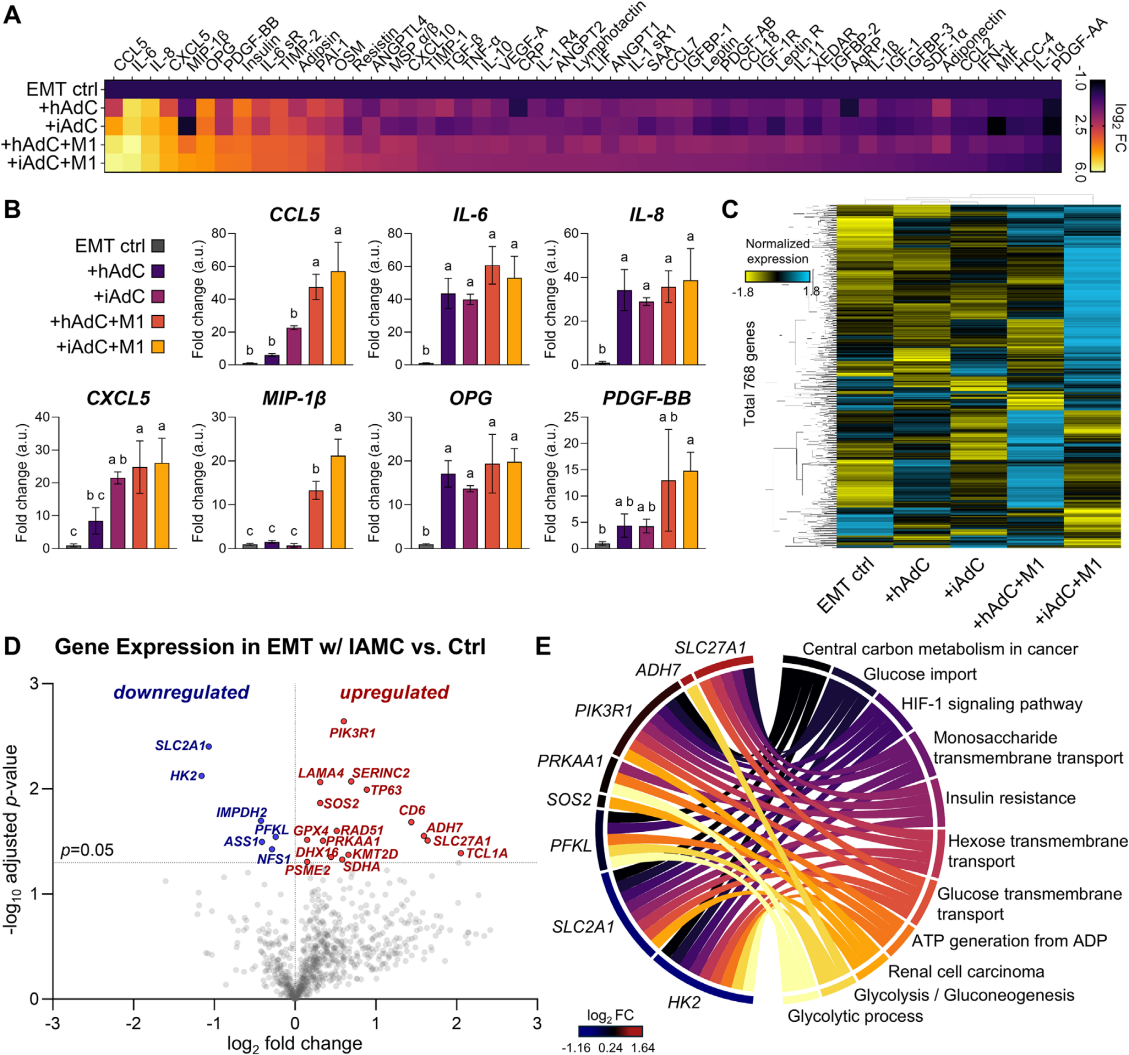
Secretome and gene expression analysis of the in vitro EMT‐IAMC co‐culture interfaces. A) Relative cytokine expression levels in the conditioned media against a panel of 52 human obesity‐specific cytokines. The color range represents the log_2_ fold expression change compared to the EMT control group. B) The top seven most pronounced cytokines in the EMT+M1+iAdC (EMT‐IAMC) group; CCL5, IL‐6, IL‐8, CXCL5, MIP‐1β, OPG, and PDGF‐BB. One‐way ANOVA followed by Tukey's multiple comparison tests. Groups assigned different letters (a, b, c) are significantly different (*p* < 0.05), while groups sharing the same letter are not significantly different. n = 3 independent conditioned media. C) Heatmap of metabolic pathway associated gene analysis in EMTs with different co‐culture conditions. Color represents the normalized expression values and lines indicate unsupervised clustering of the groups. n = 3 independent EMTs. D) Volcano plot exhibiting differentially expressed metabolic genes of EMT co‐cultured with IAMC (“+iAdC+M1”) compared to EMT only control. X‐axis displays the log_2_ fold change value and Y‐axis represents the mean expression value of ‐log_10_ adjusted *p*‐value. E) Chord graph of the top eleven activated pathways and related genes demonstrating the regulatory difference between EMT and EMT co‐cultured with IAMC group, primarily in metabolic processes.

First, conditioned medium was collected from several co‐culture groups consisting of EMT along with other cell types in the IAMC environment, and its obesity‐specific cytokine repertoire was analyzed using a sandwich‐based membrane antibody array (**Figures** **6**A; S12, Supporting Information). Notably, the top seven most pronounced panels, including chemokines (e.g., CCL5, CXCL5, and MIP‐1β) and cytokines (e.g., IL‐6, IL‐8, OPG, and PDGF‐BB), were significantly elevated in the presence of M1 macrophages and inflamed AdCs compared to those of the EMT control group (Figure 6B). Overall, the expression of the adipocyte secretomes increased with greater adipocyte inflammation and the inclusion of M1 macrophages. Specifically, compared with the EMT control, IL‐6, IL‐8, and OPG were elevated in all co‐culture groups, even including the hAdC group. CXCL5 was increased in the presence of either inflamed adipocytes or M1 macrophages, whereas CCL5 and MIP‐1β were elevated only in the M1‐containing groups. PDGF‐BB was significantly increased only in the iAdC+M1 group.

Furthermore, total RNAs were isolated from the EMTs, and expression of metabolic pathway‐associated genes was measured. The heatmap generated based on the relative expression in between groups demonstrated distinct transcriptional changes in the EMT co‐cultured with iADC + M1 (IAMC) group (Figure 6C). Among the 768 genes analyzed, 16 were significantly upregulated and 6 were downregulated in the EMT + IAMC group compared to EMT alone (Figure 6D). These differentially expressed genes (DEGs) highlight a metabolic shift in muscle tissue influenced by the inflammatory IAMC microenvironment, offering insights into muscle‐adipose crosstalk in metabolic dysfunction. The top significantly increased genes included *SLC27A1, PIK3R1, ADH7, PRKAA1*, and *LAMA4*, and key downregulated genes included *SLC2A1*, *HK2*, *IMPDH2*, and *PFKL*. Gene ontology (GO) and KEGG pathway enrichment analyses identified the eleven most significantly enriched pathways and associated genes in EMT+IAMC relative to EMT controls, revealing differential regulation of metabolic processes (Figures 6E; S13, Supporting Information).

## 3 Discussion

We established a compartmentalized human microphysiological interface coupling engineered skeletal myobundles to an inflamed adipose–macrophage niche to interrogate how obese inflammatory microenvironment perturbs muscle function via paracrine signaling (Figure 1A,B). The Transwell‐based configuration allowed independent maturation of each compartment and timed initiation of crosstalk (Figure 1C), while quantitative muscle contractile phenotyping was achieved on pillar devices with E‐Stim and force measurement from pillar deflection using a computational stiffness model. Relative to single‐gel co‐cultures or non‐compartmentalized constructs,^[^ ^21^, ^25^ ^]^ this interface offers i) spatial separation to preserve tissue identity while permitting soluble exchange, ii) temporal control over exposure windows, and iii) parallel access to media and tissue for secretome and transcriptomic analyses in addition to quantitative force readouts. In aggregate, these design features improve experimental tractability for adipose–muscle crosstalk and offer a user‐oriented experimental platform.

An important methodological contribution of this study lies in our FEA–based estimation of pillar stiffness, which provided the basis for quantitative conversion of pillar deflection into contractile force. Unlike conventional analytical methods that assumed an idealized concentrated load at the free end of the pillar,^[^ ^12^, ^24^ ^]^ our approach explicitly incorporated the experimentally observed anchorage thickness of the muscle bundle (1184 µm) together with precise pillar geometries (Figure 2G). This yielded a stiffness of 5.3 µN µm^−1^ (Figure 3C), which was consistently adopted for force estimation across all experiments. Compared to the analytical solution (2.92 µN µm^−1^, Figure S8, Supporting Information), this represents a significant difference of 45% compared to the value derived from the actual anchorage thickness. Importantly, this FEA approach was further validated against both a hanging‐mass experiment and its digital twin simulation (3.85 vs 4.12 µN µm^−1^, Figure S9, Supporting Information), confirming the robustness and reproducibility of our computational framework. By directly accounting for device geometry and tissue anchorage, this approach reduces uncertainty and enables cross‐study comparisons that have been challenging when simplified stiffness assumptions were applied. More broadly, the adoption of such FEA‐based frameworks provides a rigorous and generalizable foundation for benchmarking muscle contractility across platforms, facilitating standardization and enabling systematic exploration of how pillar design and tissue dimensions influence functional readouts.

Prior to EMT‐IAMC co‐culture, THP‐1 monocytes were differentiated to macrophages and polarized to an M1‐like phenotype. Polarization into M1 macrophages was validated by flow cytometry, showing significant increases in CD80 and CD86 in M1 cells relative to M0 macrophages, while the M2‐associated marker CD206 remained minimal, indicating negligible M2 contamination (Figures 4C,D; S3, Supporting Information). Functionally, exposure to an inflamed adipocyte was sufficient to markedly depress muscle contractility: compared with EMT‐only controls, twitch force fell by ≈86% with inflamed adipocytes and by ≈82% with the full IAMC (inflamed adipocytes + M1 macrophages) (Figure 5D). Healthy adipocytes produced a milder effect (≈39%), which was accentuated by M1 macrophage addition (≈54%). Contraction velocity exhibited concordant decrements (by 91% with inflamed adipocytes; by 84% with IAMC), with only modest, nonsignificant slowing in healthy‐adipocyte conditions (Figure 5E). These results may indicate that inflamed adipocytes are the principal drivers of contractile impairment, with macrophages plausibly acting as amplifiers in selected contexts.

Cytokine profiling of co‐cultured conditioned media revealed overall upregulation of CCL5, IL‐6, IL‐8, CXCL5, MIP‐1β, OPG, and PDGF‐BB in settings of adipocyte inflammation and with M1 macrophages (Figure 6A,B). Notably, inflamed AdCs alone increased IL‐6 and IL‐8 (Figures 4E; S10, Supporting Information), indicating that inflamed AdCs might be the primary source of these cytokines. Co‐culture with M1 macrophages further amplified and diversified the secretome. Among these, CXCL5 showed a consistent trend between our cytokine expression and muscle contractility data, which exhibited significance in the presence of either M1 macrophages or inflamed adipocytes. CXCL5, a macrophage‐secreted chemokine known to be elevated in obese human serum,^[^ ^26^ ^]^ may perpetuate muscle damage via recruitment of pro‐inflammatory leukocytes, a mechanism previously observed in acute muscle injury models.^[^ ^27^ ^]^ This parallels our IAMC system, where CXCL5 upregulation likely reflects chronic inflammation akin to obesity‐associated myopathy.

According to previous studies, CCL5, IL‐8, and MIP‐1β have been reported to promote obesity‐associated inflammation. CCL5 (RANTES), secreted from obese adipose tissue macrophages, has been linked to skeletal muscle lipid accumulation and reduced performance, shown in *Cbl‐b*‐deficient mice^[^ ^28^ ^]^ and high‐fat diet‐fed Sprague‐Dawley rats.^[^ ^29^ ^]^ The pronounced CCL5 secretion in our M1‐containing interface suggests its potential role in exacerbating inflammation‐driven muscle atrophy, mirroring in vivo obesity phenotypes. IL‐8, an adipokine expressed in obese individuals, is strongly associated with insulin resistance and adipose tissue inflammation,^[^ ^30^ ^]^ suggesting its contribution to the impaired contractility of muscle observed in our model. MIP‐1β (CCL4), elevated in individuals with obesity,^[^ ^31^, ^32^ ^]^ promotes leukocyte recruitment and pro‐inflammatory signaling, and seems primarily secreted by M1 macrophages with further amplification by inflamed adipocytes in our assay. In contrast, IL‐6, OPG, and PDGF‐BB have been reported to exert both pro‐inflammatory and protective effects in obesity depending on the context. While transient IL‐6 release can enhance glucose uptake and myogenesis,^[^ ^33^, ^34^ ^]^ prolonged exposure may promote insulin resistance and metabolic dysfunction,^[^ ^34^ ^]^ as shown in our assay. OPG has been reported to promote obesity progression via adipogenesis and inflammation in mice,^[^ ^35^, ^36^ ^]^ yet also been proposed as a potential beneficial regulator of glucose metabolism.^[^ ^37^ ^]^ PDGF‐BB has been implicated in promoting expansion of white adipose tissue via vascular remodeling in obese mice,^[^ ^38^, ^39^ ^]^ but was also strongly activated in fat‐reduced mice with regular aerobic exercise.^[^ ^40^ ^]^

Collectively, the cytokines released from co‐culture likely induced muscle dysfunction by amplifying inflammatory signaling and impairing metabolic homeostasis, as evidenced by reduced myotube integrity and contractile protein expression in co‐cultures. This is consistent with previous studies reporting that most of these cytokines are elevated in obesity and contribute to chronic low‐grade inflammation, insulin resistance, and the worsening of metabolic dysfunction, thereby exerting negative effects on health. However, their specific roles and underlying mechanisms in this complicated crosstalk condition require further investigation.

Benchmarking our secretome against Genotype‐Tissue Expression (GTEx) transcriptomes revealed both alignment and divergence across tissues. In subcutaneous adipose tissue (SAT; Figure S14A, Supporting Information), CCL5 and OPG transcripts were elevated in obesity, which is consistent with our secretion data and reports implicating CCL5/CCR5 signaling and OPG in white adipose tissue (WAT) remodeling.^[^ ^35^, ^41^ ^]^ By contrast, CXCL5 decreased in SAT mRNA yet increased in our co‐culture; this likely reflects cellular composition, as CXCL5 is enriched in macrophage/SVF rather than mature adipocytes, and circulating CXCL5 protein typically rises with obesity and falls after weight loss, matching our secretion results.^[^ ^26^, ^42^ ^]^ In visceral adipose tissue (VAT; Figure S14B, Supporting Information), transcriptional elevations in CXCL5, leptin, and IL‐6 were consistent with our secretome findings, whereas CCL4 (MIP‐1β) trended lower despite strong secretion in our system. This discrepancy is consistent with the acute, potent induction of CCL4 secretion in monocytes/macrophages by M1 polarization stimuli, a response captured within our experimental system. However, this effect may be diminished in chronic conditions, as suggested by the bulk VAT mRNA profiles.^[^ ^43^ ^]^ Additionally, in circulation (Figure S14C, Supporting Information), CCL18 was the most elevated analyte in obese patients but was not significantly altered in our model, as it is primarily produced by M2‐like macrophages, which we did not include.^[^ ^44^, ^45^ ^]^ Overall, the pattern supports a VAT‐like chemokine‐enriched profile and myokine‐mediated inflammatory crosstalk within a controlled and human‐relevant experimental platform.^[^ ^46^ ^]^

EMTs interfaced with IAMC exhibited upregulation of *SLC27A1*, *PIK3R1*, *PRKAA1*, and *LAMA4*, with concomitant downregulation of *SLC2A1*, *PFKL*, and *IMPDH2* (Figure 6D,E). The directionality of these changes, together with pathway enrichment for insulin‐resistance and glycolysis modules, indicates a shift toward fatty‐acid utilization over glycolysis, which serves as a hallmark of insulin resistance.^[^ ^47^ ^]^ This shift may arise from inflammatory mediators (e.g., cytokines and chemokines) secreted by M1 macrophages and inflamed adipocytes within IAMC, disrupting insulin signaling and promoting lipid storage. These findings underscore the utility of our 3D co‐culture in recapitulating in vivo–like metabolic perturbations and provide a tractable platform for dissecting adipose–muscle crosstalk in metabolic disease.

Among the upregulated genes, prior research has demonstrated that *SLC27A1*, the gene encoding long‐chain fatty acid transport protein 1 (FATP1), mediates mitochondrial fatty acid import in skeletal muscle cells.^[^ ^48^ ^]^ Furthermore, FATP1 overexpression in myotubes enhances fatty acid oxidation and promotes palmitate esterification into triacylglycerides.^[^ ^49^ ^]^ In addition, *SLC27A1* plays a pivotal role in the process of glucose import in response to the stimulatory effects of insulin. These suggest that EMT‐IAMC co‐culture may promote lipid and glucose flux into muscle, potentially mimicking metabolic adaptations observed in obesity. Further, GO enrichment analysis identified significant involvement of monosaccharide transmembrane transport (GO:0015749) and glucose import (GO:0046323), indicating the important role of *SLC27A1* in metabolism regulation during co‐culture. Similarly, *PIK3R1*, encoding a regulatory subunit of phosphatidylinositol 3‐kinases (PI3K) critical for insulin signaling, was enhanced. Elevated *PIK3R1* expression is linked to insulin resistance in humans, while its suppression improves insulin sensitivity,^[^ ^50^, ^51^ ^]^ glucose transport, and glucose tolerance.^[^ ^52^ ^]^ Involvement of *PIK3R1* in insulin resistance (KEGG:04931), glucose import (GO:0046323), and monosaccharide transmembrane transport (GO:0015749), implies that IAMC‐induced inflammation may disrupt muscle insulin signaling and glucose homeostasis. The glycolytic process (GO:006096) involves the *PRKAA1* gene, which is also known to enhance glycolysis and glucose transport when upregulated.^[^ ^53^ ^]^ The upregulation of *LAMA4*, a gene strongly associated with obesity in human and murine models,^[^ ^54^ ^]^ further underscores the pro‐adipogenic and inflammatory influence of IAMC on muscle.

Furthermore, among the downregulated genes, *SLC2A1* encodes GLUT1, a primary mediator of insulin‐independent glucose uptake, while *HK2* (hexokinase II) catalyzes the initial rate‐limiting step of glycolysis (glucose‐to‐glucose‐6‐phosphate conversion).^[^ ^55^ ^]^ Their suppression indicates impaired basal glucose uptake and glycolytic capacity in muscle co‐cultured with IAMC. This parallels findings in insulin‐resistant muscle, where chronic lipid exposure shifts substrate preference toward fatty acids. Similarly, reduced *PFKL* (phosphofructokinase liver type), a key glycolysis enzyme, and *IMPDH2* (rate‐limiting in guanine nucleotide synthesis), suggest compromised nucleotide metabolism and energy production, potentially exacerbating metabolic inflexibility. Moreover, the contribution of these genes to the metabolic adaptation related biological process support the dysfunction of glucose homeostasis in muscle co‐cultured with IAMC (GO:0046323 glucose import, GO:0015749 monosaccharide transmembrane transport, GO:0008645 hexose transmembrane transport, GO:1904659 glucose transmembrane transport, GO:0006757 ATP generation from ADP, GO:0006096 glycolytic process, KEGG:04066 HIF‐1 signaling pathway, KEGG:04931 insulin resistance, KEGG:00010 glycolysis/glucogenesis).

Prior MPS models have substantially advanced our understanding of skeletal muscle function.^[^ ^56^ ^]^ Although these constructs are highly effective for characterizing intrinsic muscle physiology, their relatively large dimensions can limit investigations of inter‐tissue interactions, posing practical challenges for scalability, integration with compartmentalized platforms, and precise control of crosstalk.^[^ ^57^, ^58^ ^]^ Other approaches embed vascular endothelial cells and myoblasts within a single 3D hydrogel to enable continuous, close‐range interaction.^[^ ^21^, ^59^ ^]^ However, such spatially integrated configurations can complicate co‐culture optimization, preclude independent modulation of each microenvironment, and hinder isolation of cell populations for downstream molecular analyses, often necessitating additional labeling or separation steps.

In contrast, our EMT–IAMC interfaced system mitigates these practical and analytical constraints via spatial compartmentalization in a Transwell‐compatible format. Paracrine communication occurs across a permeable membrane, affording spatiotemporal control over co‐culture initiation and facile sampling for transcriptomic or proteomic profiling. In parallel, quantitative contractile phenotyping via pillar deflection provides a direct functional readout of muscle performance under inflammatory conditions. Taken together, these features yield a versatile, analytically tractable platform for studying obesity‐associated muscle dysfunction in a physiologically relevant yet experimentally manageable context.

Despite these advantages, several limitations—and corresponding avenues for refinement—warrant consideration. The present studies probe acute exposure (days), whereas obesity is chronic;^[^ ^60^ ^]^ extending culture duration with cyclical metabolic substrates and controlled mechanical loading will be important to capture remodeling, fatigue, and recovery. The macrophage compartment, derived from THP‐1 cells, could be complemented by primary human monocyte‐derived macrophages and donor‐matched adipocytes (lean/obese; visceral/subcutaneous) to strengthen translational relevance.^[^ ^61^ ^]^ Our co‐culture system lacks adaptive immune cells, such as T and B lymphocytes, which help drive obesity‐related adipose inflammation.^[^ ^62^ ^]^ Studies have demonstrated that CD4^+^/CD8^+^ T cells and B cells accumulate in obese adipose tissue, releasing cytokines and chemokines that influence macrophage polarization and metabolic dysfunction.^[^ ^63^ ^]^ Incorporating these adaptive lymphocytes would enhance the physiological relevance of our model. Moreover, because Transwell diffusion lacks interstitial flow and vascular transport, incorporating perfused or vascularized modules could better shape gradients and pharmacologic delivery.^[^ ^64^ ^]^ Force measurements were conducted under a single stimulation regimen; further characterization, such as measuring force over a range of frequencies and including fatigue assays, might also be useful.^[^ ^13^ ^]^ Finally, despite our attempts to accurately account for anchorage geometry and pillar stiffness measurement could be further improved through standardization of tissue anchorage.

## 4 Conclusion

In this study, we developed an interfaced human cell‐based MPS that models impaired skeletal muscle contractility within an inflamed obese microenvironment. A distinctive advantage of our system lies in its modular and on‐demand construction of the co‐culture interface, which allows independent preconditioning of each tissue type, temporal control over the initiation of paracrine signaling, and increased flexibility in sampling for downstream genetic analyses. By integrating EMTs derived from human iPSC myoblasts with an IAMC composed of TNF‐α‐treated adipocytes and M1‐polarized macrophages, our platform recapitulated the pathological crosstalk between skeletal muscle and inflamed adipose tissue observed in the obese microenvironment. Through quantitative analysis of muscle contraction force and velocity using micropillar devices with FEA‐based stiffness estimation, we demonstrated significant reductions in muscle functionality in response to the inflammatory signals derived from IAMC. Furthermore, cytokine profiling and gene expression analysis revealed a distinct pro‐inflammatory and metabolically dysregulated environment, mirroring the molecular characteristics of obesity‐induced muscle dysfunction. These findings highlight the potential of our EMT‐IAMC interfaced system as a physiologically relevant platform for investigating muscle‐adipose tissue crosstalk in metabolic diseases. Moving forward, this system offers a promising tool for screening therapeutic interventions aimed at mitigating inflammation‐mediated muscle dysfunction and advancing our understanding of obesity‐related metabolic disorders.

## 5 Experimental Section

### Cell Maintenance and Differentiation

HiPSCs (Alstem, iPS11) were expanded on hESC‐qualified Matrigel‐coated 6‐well plates using mTeSR Plus medium (StemCell Technologies, 100–0274). Matrigel coating was performed by incubating 1 mL of Matrigel solution (Corning, 354277; 2% v/v in KO DMEM/F12 from Gibco) at room temperature (RT) for 1 h, followed by aspiration of the solution. A 5 µm Y‐27632 Dihydrochloride (Rock inhibitor; StemCell Technologies, 72308)‐supplemented mTeSR medium was used for the first 16 h of iPS11 incubation and then replaced with fresh culture media. Upon reaching 60% confluency, the cells were split using ReLeSR dissociation reagent (StemCell Technologies, 100–0483) or cryopreserved until use.

hiPSC‐derived myoblasts were differentiated from iPS11 following the manufacturer's instructions for the skeletal muscle differentiation medium (AMSBIO, SKM‐KITM). Briefly, iPS11 cells were dissociated with Accutase, centrifuged at 200×g, seeded into collagen‐coated flasks (Thermo Fisher, 132707) at a density of 5000 cells cm^−^^2^, and differentiated into myogenic precursors using induction medium for 6 d (Stage I, Figure S1, Supporting Information). The cells were then dissociated with 0.05% Trypsin‐EDTA (Gibco, 25300‐054), centrifuged at 200×g, seeded into collagen‐coated flasks at the same density, and differentiated into myoblasts using myoblast medium for 4 d (Stage II, Figure S1, Supporting Information). After differentiation, the hiPSC‐derived myoblasts were cryopreserved until use in device experiments.

AdCs were differentiated from ADSCs (Lonza, PT‐5006) according to the manufacturer's instructions for the adipocyte differentiation medium (Zenbio). Briefly, ADSCs expanded with growth medium (Lonza, PT4505) were dissociated with 0.05% Trypsin‐EDTA, centrifuged at 200×g, and seeded into 24‐well plates (60000 cells/well; Falcon, 353047). After 3 h of incubation, differentiation was initiated by replacing the growth medium with preadipocyte differentiation medium (Zenbio, DM‐2), and the cells were cultured for 12 d with medium replenishment every 4 d (Figure S2, Supporting Information). ADSC passages between 4 to 6 were used.

M1 macrophages were differentiated from THP‐1 monocytes as previously described.^[^ ^65^ ^]^ Briefly, BFP‐labeled THP‐1 cells were expanded with RPMI‐1640 (Gibco, A1049101) supplemented with 10% v/v FBS (Gibco, 26140079), centrifuged at 500×g, and differentiated into M0 macrophages by incubating with 150 nm Phorbol 12‐myristate 13‐acetate (PMA; StemCell Technologies, 74042)‐supplemented growth medium for 24 h, followed by a 24 h incubation in fresh medium (Figure S3, Supporting Information). The cells were then polarized into M1 macrophages by incubating with 20 ng mL^−1^ IFN‐γ (Peprotech, 300–02) and 10 pg mL^−1^ Lipopolysaccharides (LPS; Sigma, L2630) for 24 h. M2 macrophages were differentiated from the M0 macrophages by incubating with 20 ng mL^−1^ IL‐4 (Peprotech, 200–04) and 20 ng mL^−1^ IL‐13 (Peprotech, 200–13)‐supplemented growth medium for 72 h. For further experiments, M1 macrophages were dissociated with Accutase (StemCell Technologies, 7920) for 5 min at 37 °C and collected with a cell lifter (CellTreat Scientific, 229306).

All cells were maintained in a 37 °C, 5% CO_2_ incubator.

### Pillar Device Fabrication

The test device, consisting of two flexible pillars with wide caps in the gel‐casting region, was designed using a commercial CAD program (SolidWorks, Dassault Systèmes) (Figure S4, Supporting Information). A positive replica incorporating the designed features was fabricated with a 5× objective lens and UpPhoto Resin on a NanoOne 2‐photon polymerization 3D printer (UpNano) (Figure S5, Supporting Information). This process was partially conducted at MIT. Nano APT Facilities. To prevent resin warping during 3D printing, the glass substrate was pre‐functionalized by rinsing with isopropyl alcohol (IPA; VWR, 470157–450), exposing it to air plasma for 90 s, silanizing with 100 mm 3‐(Dimethylchlorosilyl)propyl methacrylate (Sigma, 64145) and 97.8 mm toluene (Sigma, 179418) in ethyl alcohol for 1 h at RT, and then rinsing again with IPA and distilled water.^[^ ^66^ ^]^ The 3D‐printed positive replica was treated with air plasma for 90 s and Trichloro(1H,1H,2H,2H‐perfluorooctyl)silane (Sigma, 448931) in a desiccator for 4 h. A PDMS (Dow, SYLGARD 184 Silicone Elastomer Kit) negative replica was fabricated by pouring unpolymerized PDMS (10:1 w/w base:curing agent) onto the positive replica, degassing air bubbles, and curing the PDMS in a 60 °C oven for 4 h. Using the negative replica, a positive PDMS pillar device was fabricated by repeating the process of the negative replica. Finally, holes were created in the PDMS block with an 8 mm diameter biopsy punch, and notches were added using a 4 mm diameter biopsy punch. The device was then sonicated in 70% v/v ethyl alcohol for 15 min, autoclaved for 15 min, and stored at RT until use.

### On‐Device Formation and Differentiation of 3D Muscle Tissue

A 5% w/v Pluronic F‐127 (Sigma, P2443) solution was prepared by fully dissolving the powder in PBS (Gibco, 10010–023) overnight at 4 °C and filtering it through a 0.2 µm‐pore membrane. The solution was added to the casting region of RT‐equilibrated devices, and the devices were then incubated at 37 °C for 2 h. After incubation, they were washed with distilled water and fully dried at RT. Subsequently, hiPS‐myoblast suspensions were prepared by dissociating the cells with 0.05% Trypsin‐EDTA, centrifuging at 200×g for 5 min, and resuspending in Skeletal Muscle Myoblast Medium (AMSBIO, SKM02). A gel‐cell mixture was made by mixing CellMatrix (Component A of porcine collagen gel culturing kit; Nitta Gelatin Inc., 638–00781), reconstitution buffer (Component C of the kit), GFR‐Matrigel (Corning, 356231), and the cell suspension on ice. The final mixture had a cell density of 1×10^7^ cells mL^−1^, a collagen gel concentration of 2.16 mg mL^−1^, and 10% Matrigel. A 48 µL aliquot of the mixture was added to the casting region of the device, which was then placed in a sterilized humidity chamber. After gel seeding, the humidity chamber was set at 37 °C for 30 min to facilitate gel polymerization. The devices were then transferred to an empty 24‐well plate, and 1 mL of differentiation media (CuriBio, SKM‐MED‐IPS‐D) was carefully added to each well to avoid displacing the gel from the pillars. The differentiation media were replaced with fresh media every other day until d 10 for the control group or d 8 for the co‐culture group, respectively.

### Immunofluorescence Staining and Imaging

To enhance antibody penetration, cryosectioning of the EMT was performed when necessary (Figure S6, Supporting Information; Figure 2H,I). Briefly, the EMT was washed three times with PBS and fixed overnight at 4 °C in 4% v/v paraformaldehyde (PFA; Invitrogen, J19943.K2) prepared in PBS. Following fixation, the EMT was rinsed three times for 10 min each with 0.1% v/v Triton X‐100 (Sigma, X‐100) in PBS (PBS‐Triton), gently detached from the pillar using a tweezer, and placed in 30% w/v sucrose (Macron, S8360‐04) in PBS overnight at 4 °C. The EMT was then placed in a frozen cryomold filled with Optimal cutting temperature (OCT) compound (Sakura Finetek, 4583), fully covered with additional OCT compound, and quickly frozen using a dry ice/ethanol slurry. Cryosectioning was performed using a cryostat (Thermo Shandon Cryotome SME) at a thickness of 30 µm. The sectioned samples were mounted on slides, washed for 10 min with PBS‐Triton, and blocked with CAS‐Block Histochemical Reagent (Invitrogen, 008120) for 30 min at RT. Subsequently, the samples were incubated with the primary antibody (1:200 dilution) for 24 h at 4 °C, followed by incubation with the mixture of secondary antibody (1:200), Alexa Fluor 594 Phalloidin (1:400; Thermo Fisher, A12381), and DAPI (1:500) overnight at 4 °C. The primary antibodies were obtained as follows: Myosin 4 monoclonal antibody (MF‐20; Invitrogen, 14‐6503‐82), Sarcomeric α‐Actinin antibody (Abcam, ab9465), Anti‐Slow Skeletal Myosin Heavy chain antibody (Abcam, ab173366), and PPAR gamma monoclonal antibody (Invitrogen, MA5‐14889). The antibodies were prepared in a 40% v/v CAS‐Block solution diluted with PBS. Between antibody incubation steps, the samples were rinsed three times with PBS. Finally, the samples were covered with antifade mounting medium (Vector, Laboratories, H‐1000), sealed with a coverslip, and visualized using a confocal laser scanning microscope (Olympus FLUOVIEW FV1000) with 10× and 60× objectives. If required, Z‐stack images were acquired with step sizes of 4.5 and 0.44 µm for the respective objectives. For samples where cryosectioning was not required, the step was omitted, and the staining procedure was performed as described.

### Analysis of EMT Shape and Nuclear Fusion Index

During on‐device differentiation, on days 2, 6, and 10, top‐view images of the EMT were captured using a smartphone mounted on a microscope in place of the eyepiece. The images were analyzed using ImageJ software (NIH, Bethesda, USA). After cropping a region that included the EMT, the image was binarized, and shape factors (area, width, and aspect ratio) of the EMT were measured using the Fit Ellipse tool. Eccentricity of the EMT was calculated using the following metric: √(1‐1/aspect ratio^2^).

On day 10, after fixing the EMT with PFA, the sides of the device were cut, and the device was mounted on the microscope stage to capture a side‐view image of the EMT. The tissue thickness and anchorage length of the EMT were measured using ImageJ. Cryosectioned EMT samples and confocal images were prepared as described above to analyze the nuclear fusion index of myotubes, defined as the number of nuclei within multinucleated myotubes (>2 nuclei) divided by the total number of nuclei. Nuclei exhibiting fragmentation or lacking α‐actinin expression were excluded from the analysis.

### Measurement of Young's Modulus of PDMS

The Young's modulus of the PDMS material was independently measured by AFM indentation using a JPK NanoWizard4 system equipped with a PR‐T300 probe (Probes) (Figure S7, Supporting Information). The actual spring constant of each cantilever was calibrated using the thermal noise method prior to measurement. Force–distance curves were acquired at multiple locations on the PDMS surface under ambient conditions, and the indentation depth was determined from the contact point in the approach curve. The Young's modulus was extracted by fitting the approach curve to the Hertz contact model for a conical indenter,^[^ ^67^, ^68^ ^]^ using a Poisson's ratio of 0.495 for PDMS.^[^ ^69^ ^]^ At least 9 measurements were performed to obtain the average modulus and standard deviation.

### Computational Estimation of Pillar Stiffness

The pillar stiffness was determined by using FEA. Based on the measured dimensions of pillar and anchorage thickness with a brightfield microscope (Figure S5E, Supporting Information; Figure 2G), the pillar structure was computationally reconstructed using 3D design software SolidWorks. Pillar deflection, depending on the anchorage thicknesses where muscle force was applied, was analyzed using COMSOL Multiphysics (Version 6.2). Pillar stiffness, *k*, defined as the ratio of the applied force to the displacement of the pillar top, was then calculated from the linear FEA solution. The parameter values of PDMS used in the FEA were: Young′s modulus(E) = 1.42 MPa measured with AFM; Poisson′s ratio(v) = 0.495^26^ and *density* (ρ) = 1030 kg m^−3^. The bottom end of the pillar was assumed to be rigidly attached to the substrate.

### Measurement of Pillar Stiffness via the Hanging‐Mass Method

Pillars were mounted horizontally with one end rigidly fixed, and a thread (with approximate thickness of 270 µm) was looped around the right before the pillar's cap to prevent slippage during loading (Figure S9, Supporting Information). Static loads were applied by suspending plastic beads of known mass (0, 173, 224, and 428 mg) from the thread, with the total suspended mass measured on a precision balance and converted to force using *F*  =  *mg* (*g* = 9.81 m ^−1^s^2^). Each loading condition was held for at least 5 s to minimize oscillations, and images were captured at fixed magnification with a 4 mm scale bar. The vertical displacement of the pillar's free end relative to the unloaded position was measured in ImageJ using the “Set Scale” function for calibration. The effective stiffness was calculated from the 1/slope of the force‐displacement curve obtained by linear regression constrained through the origin.

### Creation of IAMC‐In‐Well and Merging with EMT

After 12 days of differentiation of ADSCs, AdCs were incubated with AdC Maintenance Medium (Zenbio, AM‐1) supplemented with 2 ng mL^−1^ TNF‐α (Peprotech, 300–01A) for 24 h, followed by rinsing with warm PBS. Adipocyte viability across the multiple concentrations of TNF‐α was confirmed using CellTiter‐Fluor cell viability assay (Promega, G6080) according to manufacturer's instructions. M1 macrophages were then reconstituted with Maintenance Medium supplemented with 50 ng mL^−1^ M‐CSF (Peprotech, 300–25) and added to the AdC well (60000 cells/well). The co‐culture was maintained for 3 days to generate IAMC. Finally, EMTs on 3 µm‐pore Transwell inserts (Falcon, 353096) were transferred to IAMC wells with fresh muscle differentiation medium for an additional 2 d of co‐culture.

### Human Adipokine Luminex Assay for Adipokine Secretion Profiling

The Milliplex Human Adipokine Magnetic Bead Panel (MilliporeSigma) was used according to the manufacturer's protocol on a Luminex FlexMAP 3D instrument (Figure S10, Supporting Information). Briefly, magnetic capture beads were vortexed and diluted 1:1 in assay buffer. A 16‐point standard curve was generated via serial two fold dilution in assay buffer. 12.5 µL of diluted beads was added to each well of a 384‐well plate. Then, 12.5 µL of standards or samples were added, followed by 25 µL of assay buffer for a final volume of 50 µL. The plate was sealed and incubated overnight at 4 °C with shaking at 600 rpm.

The plate was washed three times with 75 µL wash buffer using a magnetic plate washer (Biotek). Subsequently, 12.5 µL of detection antibody (diluted 1:1 in assay buffer) was added, and the plate was incubated for 1 h at room temperature with shaking. Next, 12.5 µL of streptavidin‐phycoerythrin (SAPE, diluted 1:1) was added, followed by a 30 min incubation with shaking. A final wash step was performed as described. After resuspension in 75 µL sheath fluid and incubation with shaking for 5 min, the plate was analyzed on the Luminex FlexMAP 3D system.

### Electrical Stimulation and Muscle Contractility Calculation

A customized setup for E‐Stim was fabricated as follows (Figure S11, Supporting Information): A PDMS holder, designed to secure the pillar device in the correct position and perforated with 8 and 4 mm diameter biopsy punches, was placed in a well of a 24‐well plate. The lid was perforated with a 1 mm punch to insert two 0.5 mm‐diameter platinum electrodes (Thermo Fisher, 010286‐BU), which were soldered to tin‐coated copper wire extensions (McMaster‐CARR, 8871K42). The setup was pre‐sterilized using a UV ramp or 70% ethyl alcohol. On day 10 of muscle differentiation, the device was transferred to the holder of the E‐Stim setup, and 1 mL DMEM (Gibco, 11965092) was added to the well. After covering the setup with the lid, the wires were connected to a waveform generator (Siglent, SDG1032X) with a BNC‐to‐clip cable. Upon applying an electric signal with repetitive square waves at 9 V_p‐p_ (electrical field strength of 1 V mm^−1^), a 20% duty cycle, and 1 Hz, the displacement of the pillar's top surface was monitored using a brightfield microscope with a 4× objective, at a video acquisition rate of 12.5 frames/s. Pillar displacement was measured from the video using ImageJ, and the maximum displacement value was used to calculate muscle force generation by multiplying it by the simulated pillar stiffness of 5.30 µN µm^−1^. Muscle contraction velocity was calculated by dividing the displacement by the corresponding time interval.

### M1 Recruitment Around the Inflamed AdC

After the E‐Stim assay, the IAMC well was fixed as mentioned above and counterstained with BODIPY 493/503 (Invitrogen, D3922) and Phalloidin to visualize AdC lipid droplets and the F‐actin of all cells, respectively. Fluorescence images acquired using an epifluorescence microscope were analyzed to count the number of BFP‐labeled M1 macrophages in contact with clusters of AdC lipid droplets.

### Cytokine Array

Conditioned media samples from the EMT‐IAMC system were analyzed using a human obesity cytokine array (RayBiotech, AAH‐ADI‐1‐8) according to the manufacturer's instructions. In brief, the 3D EMT and IAMC interface was incubated with differentiation media from d 8 to d 10, after which the conditioned media were harvested and stored at −70 °C until use. Multiple membranes were incubated with blocking solution, conditioned media, biotinylated antibody cocktails, and labeled streptavidin. The resulting membranes were then imaged using a chemiluminescence imaging system (ChemiDoc, Bio‐Rad Laboratories). Spot signal densities were quantified using the Protein Array Analyzer plugin in ImageJ software^[^ ^70^ ^]^ and normalized using Positive and Negative Spot Control densities on each membrane. Finally, log_2_ fold changes in signal densities for each group, relative to the EMT control, were calculated.

### EMT Gene Expression

Total RNA was extracted from EMTs using the Monarch Total RNA Miniprep Kit (New England Biolabs, T2010S) according to the manufacturer's protocol. The RNA yield was quantified using the NanoDrop 8000 Spectrophotometer (Thermo Fisher Scientific) and adjusted to a concentration of 10 ng µL^−1^ using Molecular Biology Grade Water (Corning, 46‐000‐CM). The RNA samples were then subjected to the NanoString nCounter Pro Analysis System, specifically the nCounter XT Metabolic Pathways Panel, in accordance with the manufacturer's instructions. The gene expression data were subsequently processed using the nCounter advanced analysis software. NanoString's standard normalization uses a combination of positive control normalization, which uses synthetic positive control targets, and CodeSet content normalization, which uses housekeeping genes, to apply a sample‐specific correction factor to all the target probes within that sample lane. The relative expression of genes was normalized to a set of internal reference genes (housekeeping genes) from the panel, including *ABCF1, AGK, COG7, DHX16, DNAJC14, EDC3, FCF1, G6PD, MRPS5, NRDE2, OAZ1, POLR2A, SAP130, SDHA, STK11IP, TBC1D10B, TBP, UBB, and USP39*. The data for GO analysis, C2: Curated gene sets collection in MSigDB for KEGG pathway, and C5: GO gene sets collection in MSigDB for Biological Process (BP), Cellular Component (CC), and Molecular Function (MF) were utilized. The chord graph of the gene analysis was visualized using DAVID, Cytoscape software (version 3.10.1) with the ClueGO application, and RStudio running R (version 4.2.2) with the Circos module.

### Flow Cytometry

Cells were resuspended in cell staining buffer (BioLegend, 420201). Cell suspensions were blocked for 5 min at 4 °C with Human TruStain FcX (BioLegend, 422301) in cell staining buffer (1:100), and incubated for 30 min with a mix of antibodies binding CD80 (1:100; BioLegend, 751732), CD86 (1:100; BioLegend, 305438), CD206 (1:100; BioLegend, 321104), and Zombie NIR fixable viability kit (BioLegend, 423105) at 4 °C. Stained cells were then fixed with fixing buffer (Invitrogen, 00‐5523‐00) overnight. The next day, the intracellular marker CD68 (1:100; BioLegend, 333810) was incubated for 30 min. Flow analysis was performed on a FACSymphony A5 (BD Biosciences).

### Human RNAseq Analysis

Human gene expression data used in this study were obtained from the GTEx Portal under dbGaP accession numbers phs000424.v10.pht002742 and phs000424.v10.pht002741, accessed on February 18, 2025. Raw count data from whole blood and skeletal muscle tissues were extracted for downstream analysis. Subjects were stratified based on body mass index (BMI). Individuals with a BMI < 30 were classified as non‐obese, while those with a BMI ≥ 30 were classified as obese. Whole blood n: obese 223 and non‐obese 580, and skeletal muscle n: obese 234 and non‐obese 584, visceral adipose n: obese 170 and non‐obese 417, adipose subcutaneous n: obese 211 and non‐obese 503. Differential gene expression (DGE) analysis was performed using the DESeq2 pipeline (DESeq2 1.48.1, R version 4.4.0).^[^ ^71^ ^]^ The design formula included BMI category as the primary variable of interest, while adjusting for age, sex, and cohort to account for potential confounding effects. Genes with an adjusted *p*‐value (FDR) < 0.05 were considered significantly differentially expressed.

### Statistical Analysis

All measurements were reported as averages ± standard deviations unless otherwise specified. Measurements were compared using the two‐sample *t*‐test when comparing two conditions, or one‐way or two‐way analysis of variance (ANOVA) with the Tukey multiple comparison test when applicable. Statistical analysis was performed using Prism Software (GraphPad). All tests yielding *p* < 0.05 were considered to be statistically significant: *, **, ***, and **** indicate *p*‐value < 0.05, 0.01, 0.001, and 0.0001 between the conditions, respectively, and n.s. Indicates statistically no significant difference between the conditions. In some graphs, compact letter display was used to enhance the visualization of multiple pairwise comparisons. Groups assigned different letters (a, b, c) were significantly different (*p*‐value < 0.05), while groups sharing the same letter are not significantly different.

## Conflict of Interest

Roger D. Kamm is a co‐founder of AIM Biotech, a company that markets microfluidic technologies, and receives research support from Amgen, Abbvie, Boehringer‐Ingelheim, Novartis, Daiichi‐Sankyo, Roche, Takeda, Eisai, EMD Serono, and Visterra.

## Author Contributions

S.K. and T.C. contributed equally to this work. S.K. and T.C. designed the device, conducted the experiments, and analyzed the data. Z.W. provided the cell sources and supported the experiments. J.K., E.C.K., T.O., and J.S.J. contributed to data analysis. Z.L., L.A.R.I., R.S., S.S., H.M., S.R., G.L., and M.P. supported experimental procedures and data interpretation. C.R.W. and R.D.K. supervised the overall project. All authors contributed to the writing and revision of the manuscript.

## Supporting information



Supporting Information

Supplemental Video 1

Supplemental Video 2

## Data Availability

The data that support the findings of this study are available from the corresponding author upon reasonable request.
