# Supplementary material for: A Microphysiological Interface of Skeletal Myobundles and Inflamed Adipose Tissue for Recapitulating Muscle Dysfunction in an Obese Microenvironment
Source: Adv Healthc Mater. 2025 Nov 10;15(5):e02711. doi: 10.1002/adhm.202502711 (PMC12864593; doi:10.1002/adhm.202502711)
Supplement: Supplementary file 1 — Supporting Information [file ADHM-15-0-s002.docx]

Supporting Information for

**A Microphysiological Interface of Skeletal Myobundles and Inflamed Adipose Tissue for Recapitulating Muscle Dysfunction in an Obese Microenvironment**

Seunggyu Kim^a,b,k+^, Tianxin Cao^c,+^, Zhengpeng Wan^d^, Jaesang Kim^e^, Zhuxuan Li^f^, Legairre A. Radden II^g^, Rakesh Santhanam^h^, Eunkyung Clare Ko^a^, Tatsuya Osaki^i^, Sarah Spitz^d^, Hyunmin Moon^a^, Maria Proestaki^d^, Seokbeom Roh^j^, Gyudo Lee^j,b,k^, Jessie S. Jeon^e^, Curtis R. Warren^c,*^, and Roger D. Kamm^a,d,*^

*^a^Department of Mechanical Engineering, Massachusetts Institute of Technology, Cambridge, Massachusetts 02139, USA*

*^b^Department of Digital Healthcare Engineering, Korea University, Sejong 30019, Republic of Korea*

*^c^Cardiovascular-Renal Metabolic Diseases Research Department, Boehringer Ingelheim Pharmaceuticals, Inc, Ridgefield, Connecticut 06877, USA*

*^d^Department of Biological Engineering, Massachusetts Institute of Technology, Cambridge, Massachusetts 02139, USA*

*^e^Department of Mechanical Engineering, Korea Advanced Institute of Science and Technology, Daejeon 34141, Republic of Korea*

*^f^Oncology Research Department, Boehringer Ingelheim Pharmaceuticals, Inc, Ridgefield, Connecticut 06877, USA*

*^g^Immunology and Respiratory Research Department, Boehringer Ingelheim Pharmaceuticals, Inc, Ridgefield, Connecticut 06877, USA*

*^h^Computational Innovation Department, Boehringer Ingelheim Pharma GmbH & Co., Biberach an der Riss, 88400, Germany*

*^i^Picower Institute for Learning and Memory, Massachusetts Institute of Technology, Cambridge, Massachusetts 02139, USA*

*^j^Department of Biotechnology and Bioinformatics, Korea University, Sejong 30019, Republic of Korea*

*^k^Digital Healthcare Center, Sejong Institute of Business and Technology, Korea University, Sejong 30019, Republic of Korea*

+These authors contributed equally.

*Corresponding authors:

Dr. Roger D. Kamm ([rdkamm@mit.edu](mailto:rdkamm@mit.edu))

Dr. Curtis R. Warren ([curtis.warren@boehringer-ingelheim.com](mailto:curtis.warren@boehringer-ingelheim.com))

**This file includes: Figure S1 to S14.**


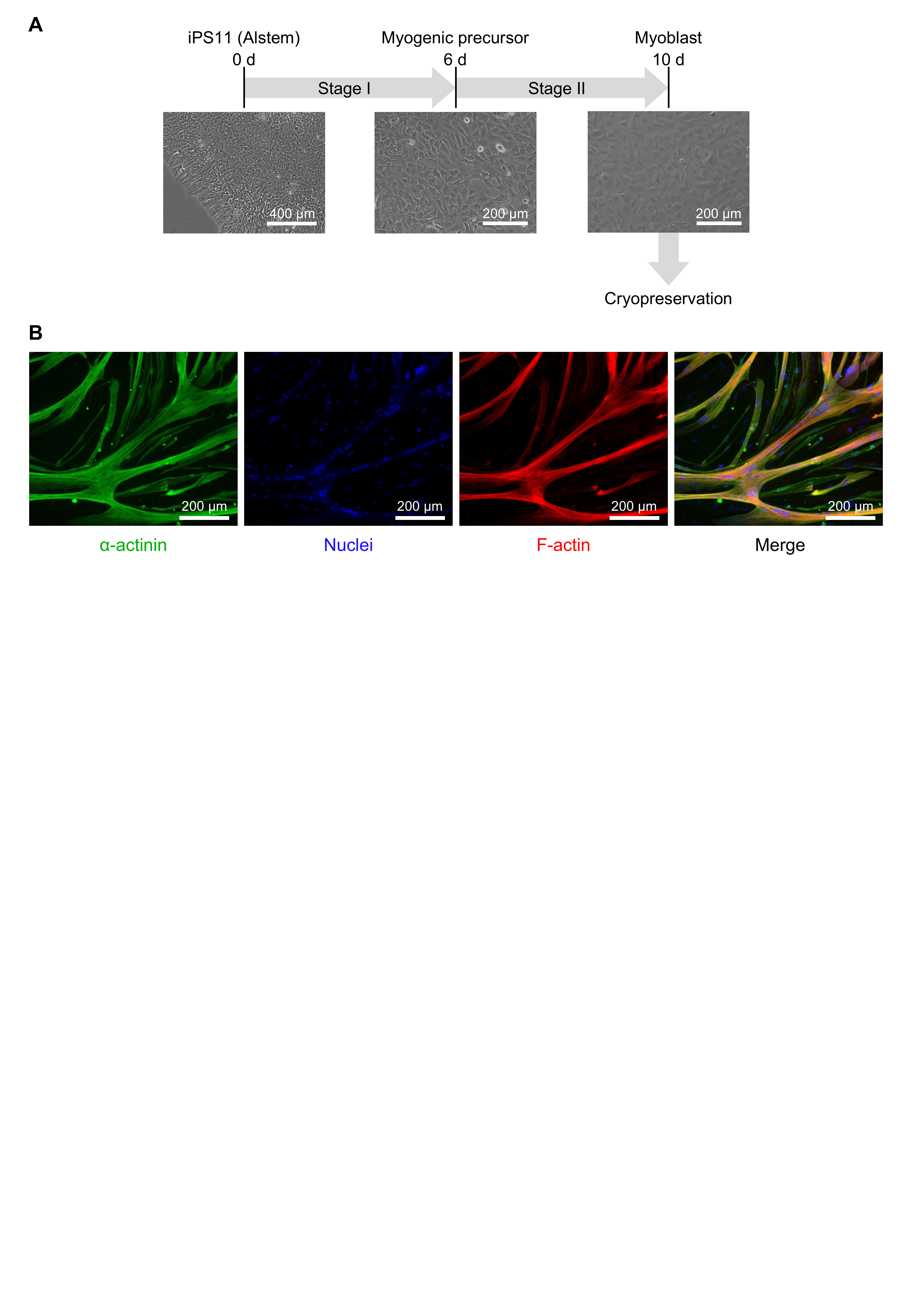


**Figure S1.** Differentiation of Alstem iPS11 into hiPS-myoblasts. (A) Images showing different stages of myoblast differentiation. (B) Immunofluorescence staining of 2D-cultured myotubes derived from hiPS-myoblasts for α-actinin expression at day 7. Green: α-actinin, blue: nuclei; red: F-actin.


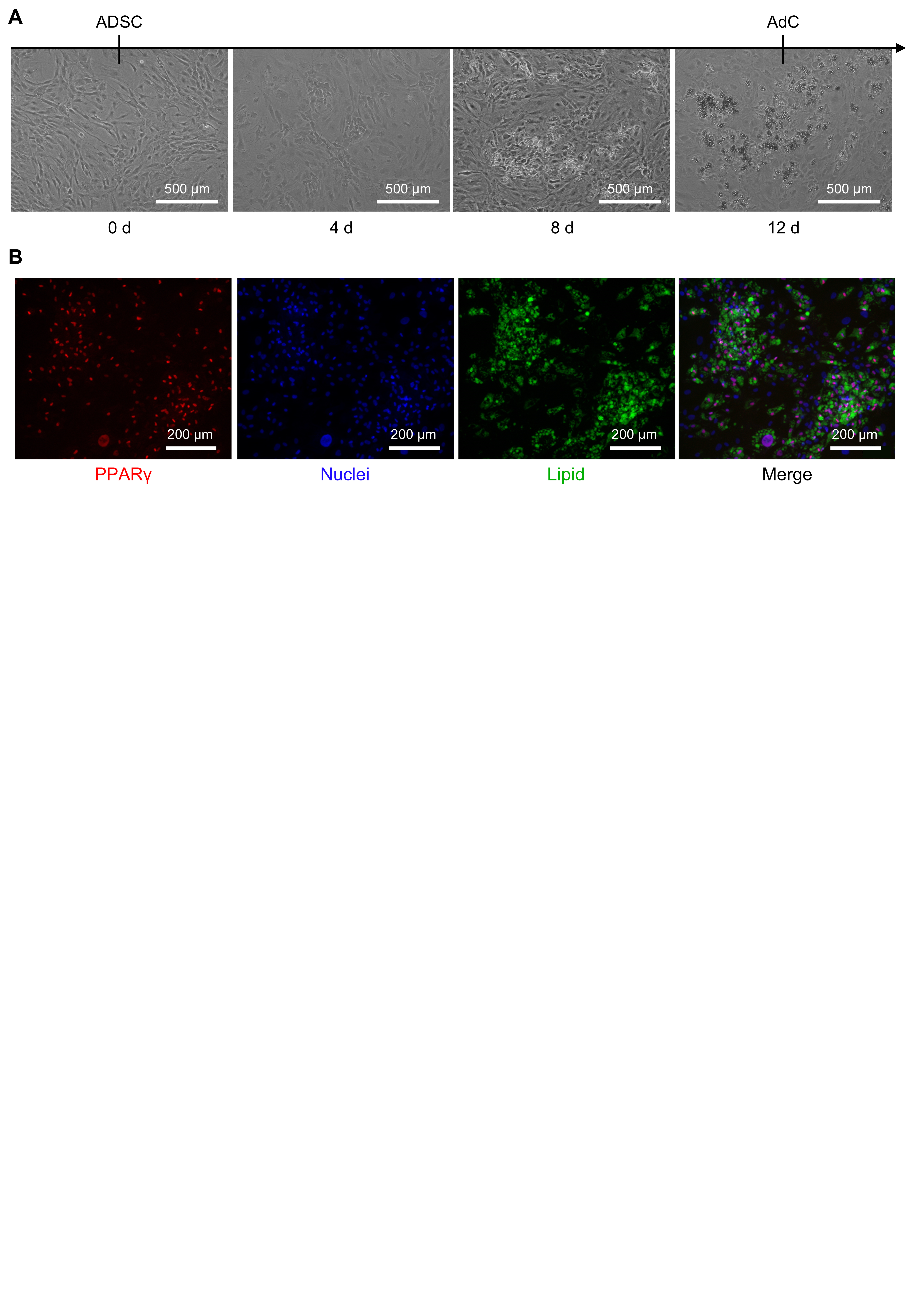


**Figure S2.** Differentiation of ADSCs into AdCs. (A) Images showing AdC differentiation over multiple days. (B) Immunofluorescence images of AdC differentiation marker. Red: PPARγ, blue: nuclei, green: lipid.


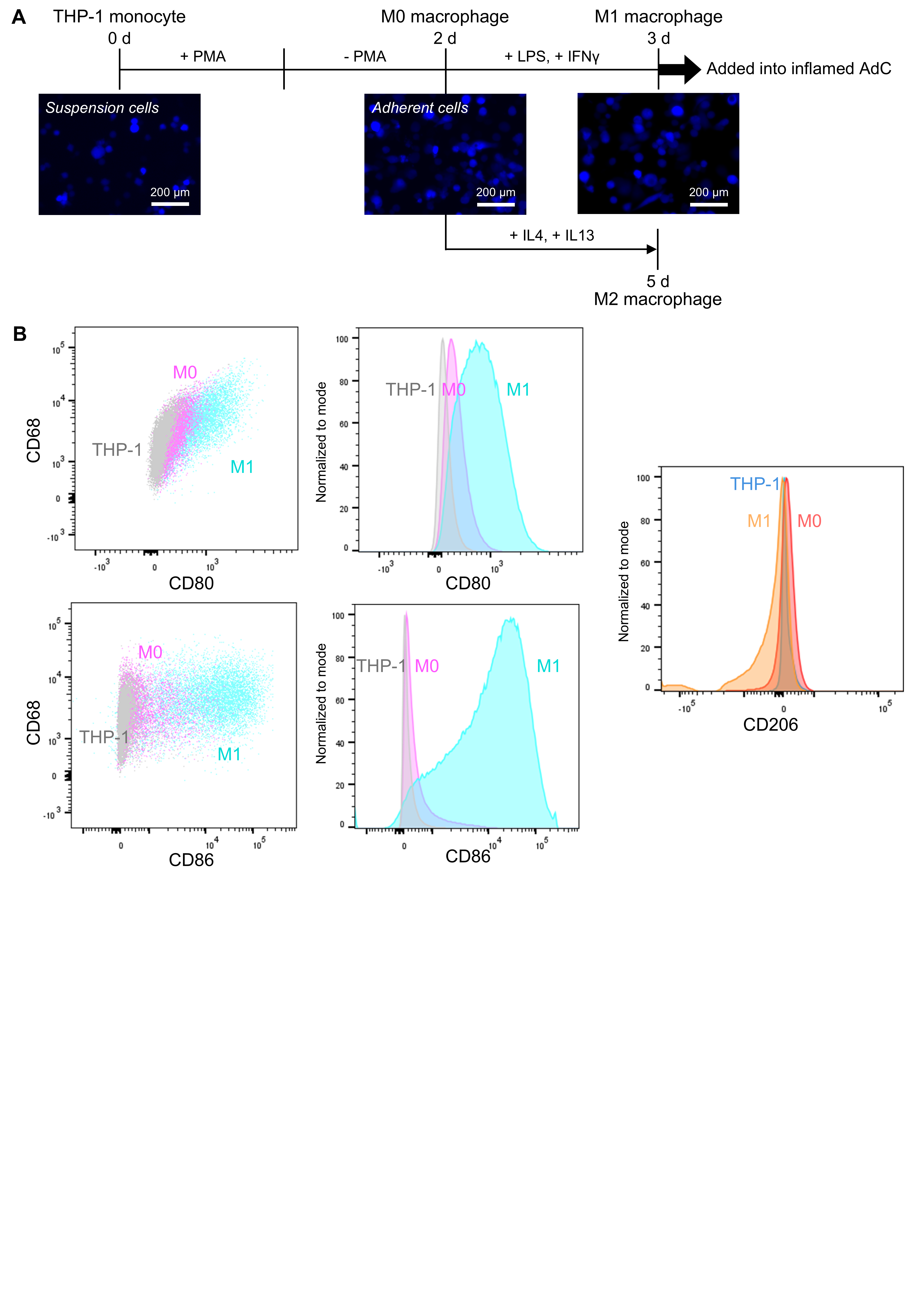


**Figure S3.** Differentiation of THP-1 monocytes into M1 or M2 macrophages. (A) Morphological changes during differentiation and polarization. (B) Flow cytometry validation of M1 macrophages with surface markers CD80, CD86, and CD206, and intracellular marker CD68 expression.


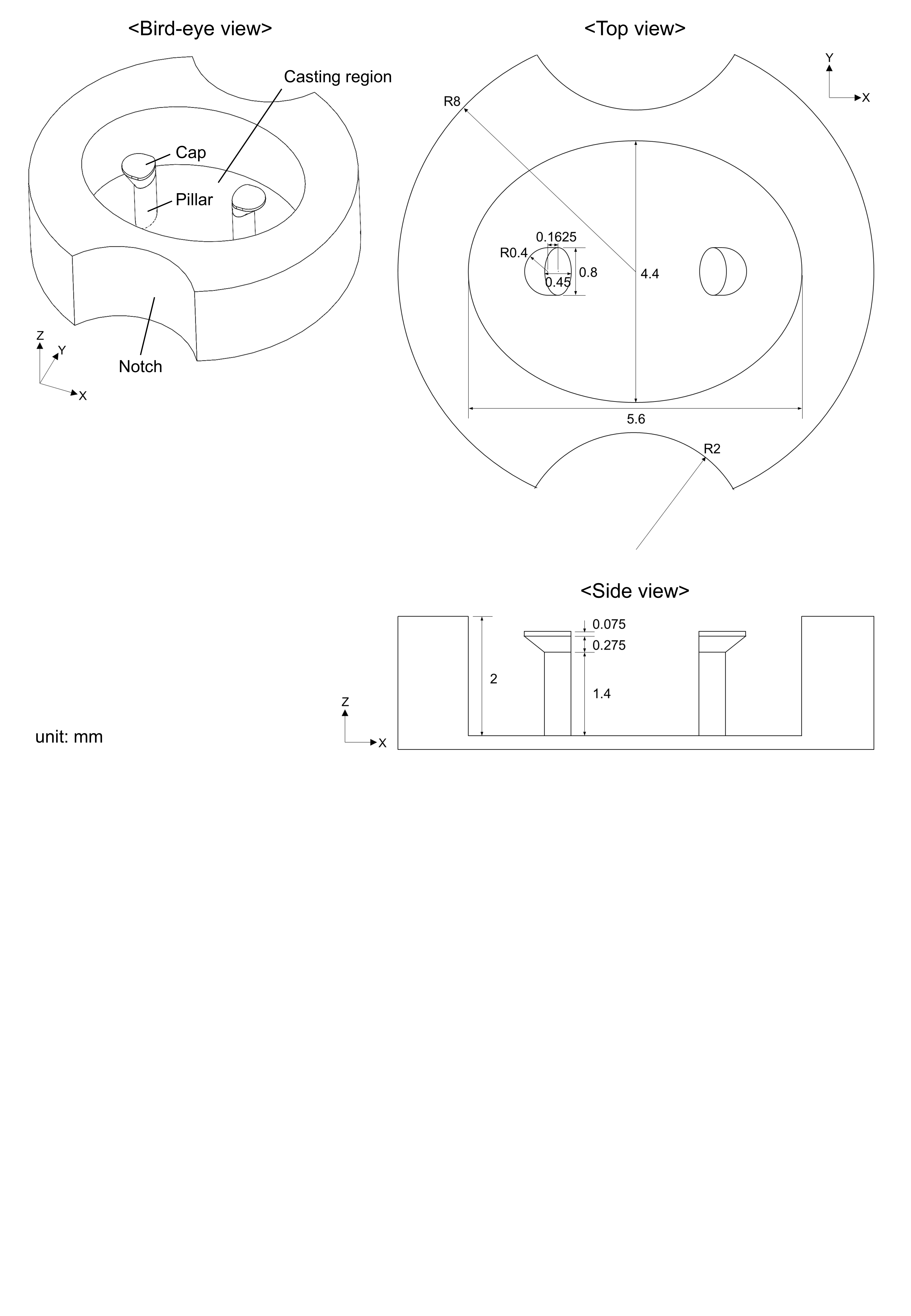


**Figure S4.** Design of the device, including pillars and the casting region.


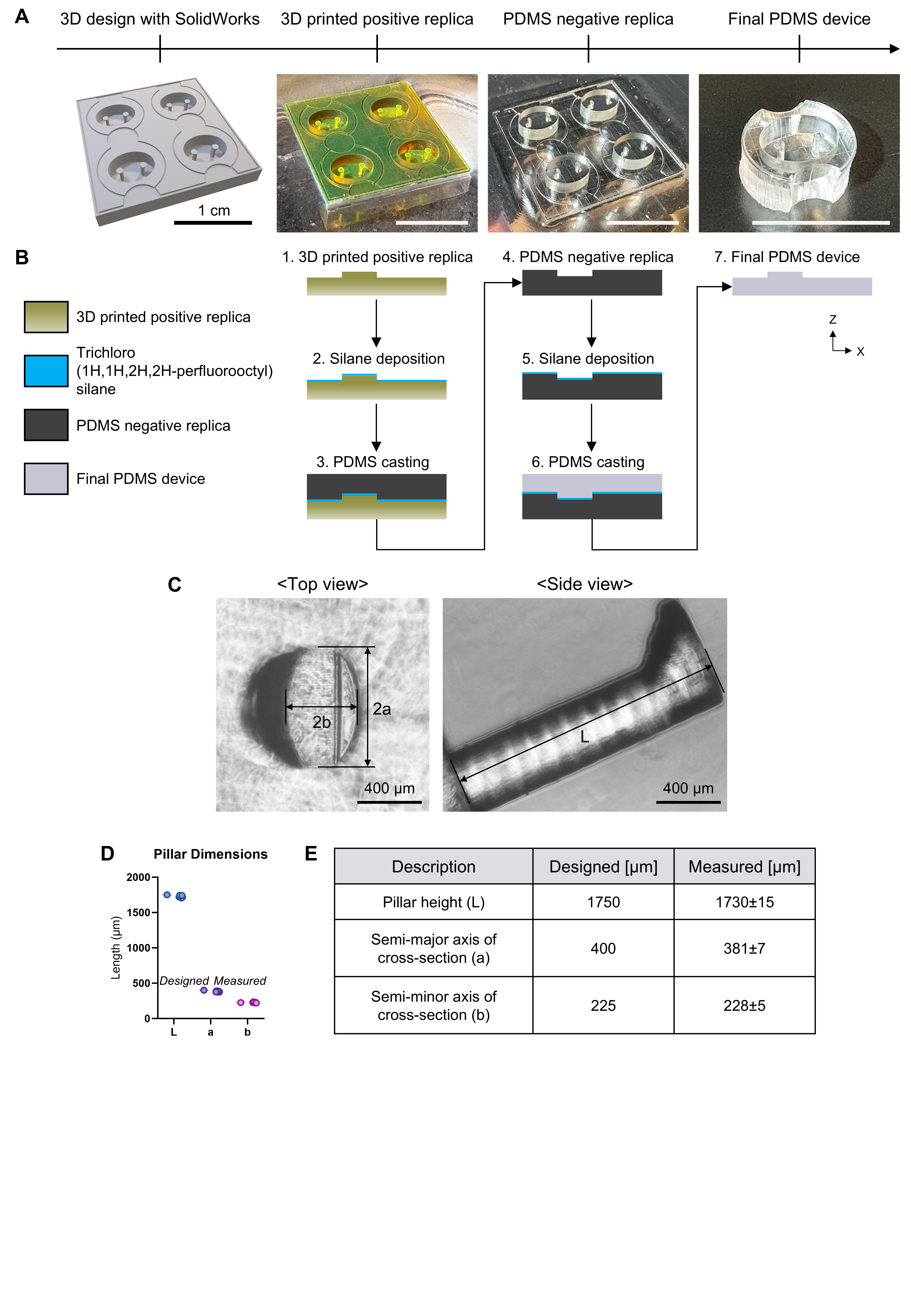


**Figure S5.** Fabrication of pillar device. (A-B) Fabrication steps with corresponding optical images and schematics. Scale bars 1cm. (C) Microscopic images of the pillar in top and side views. (D-E) Comparison of pillar dimensions between designed and measured values, including height (*L*), semi-major axis (*a*) and semi-minor axis (*b*) of the cross-section. n=7 devices.


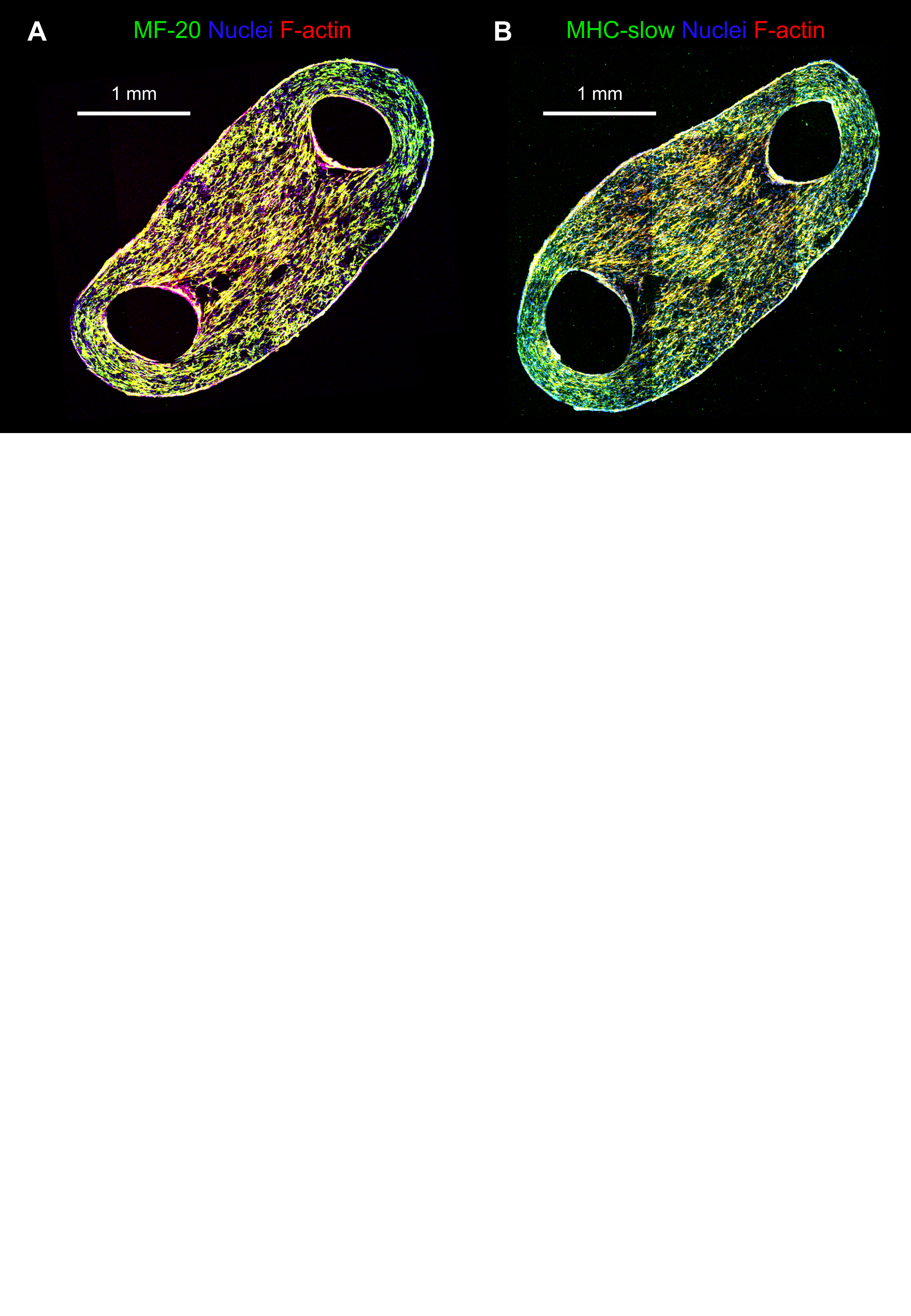


**Figure S6.** Z-projected immunofluorescence images of a 30 μm cryosectioned EMT slice stained for (A) MF-20 and (B) MHC-slow, respectively. Green: specific myotube markers; Blue: nuclei; red: F-actin.


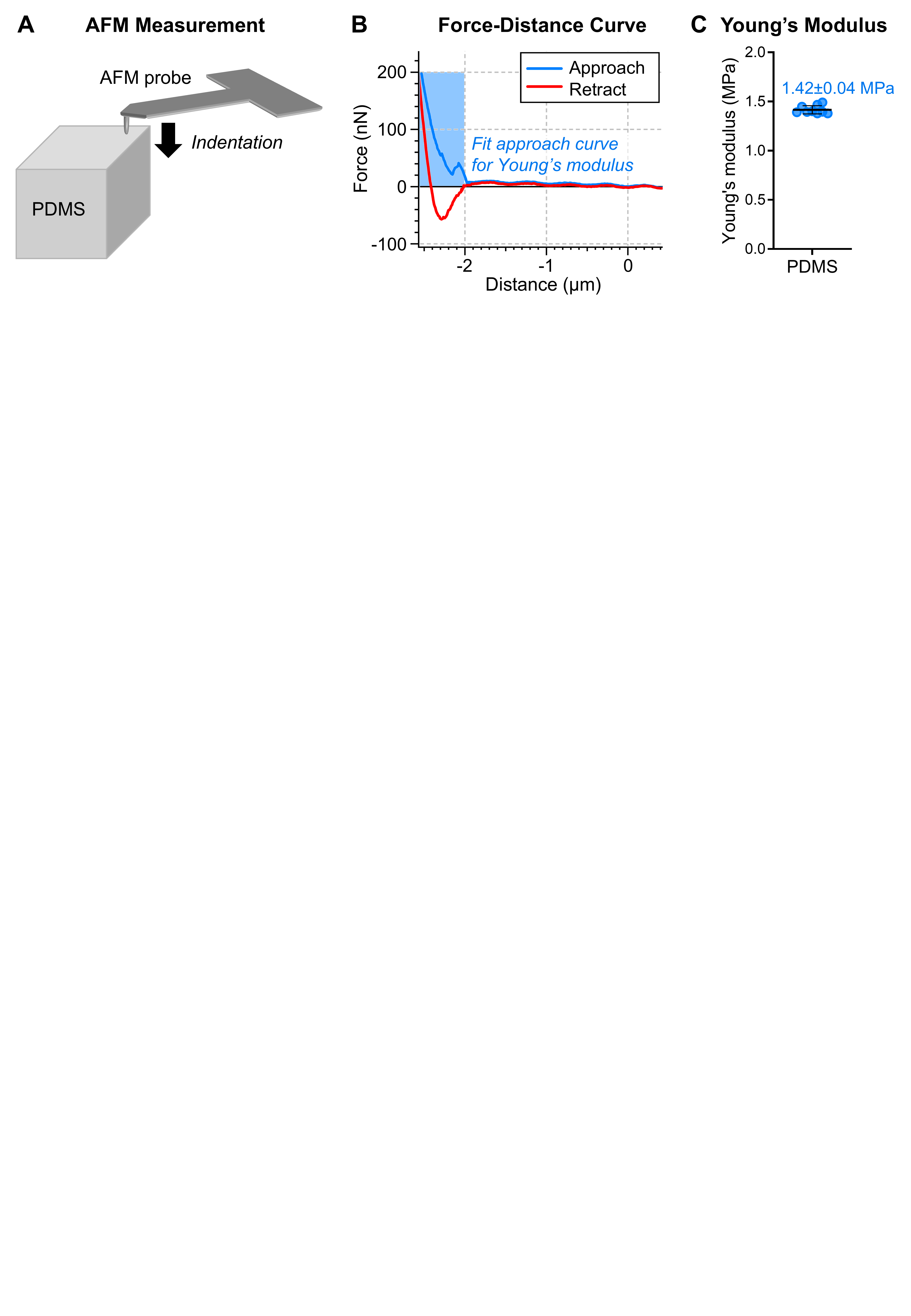


**Figure S7.** AFM-based measurement of Young’s modulus for solid PDMS. (A) Schematic of AFM measurement on a PDMS sample using a cantilever-mounted probe. (B) Representative force–distance curve showing approach (blue) and retract (red) segments. The shaded region of the approach curve was fitted to determine Young’s modulus according to the Hertz contact model. (C) Quantified Young’s modulus of PDMS, 1.42±0.04 MPa. n=9 independent samples.


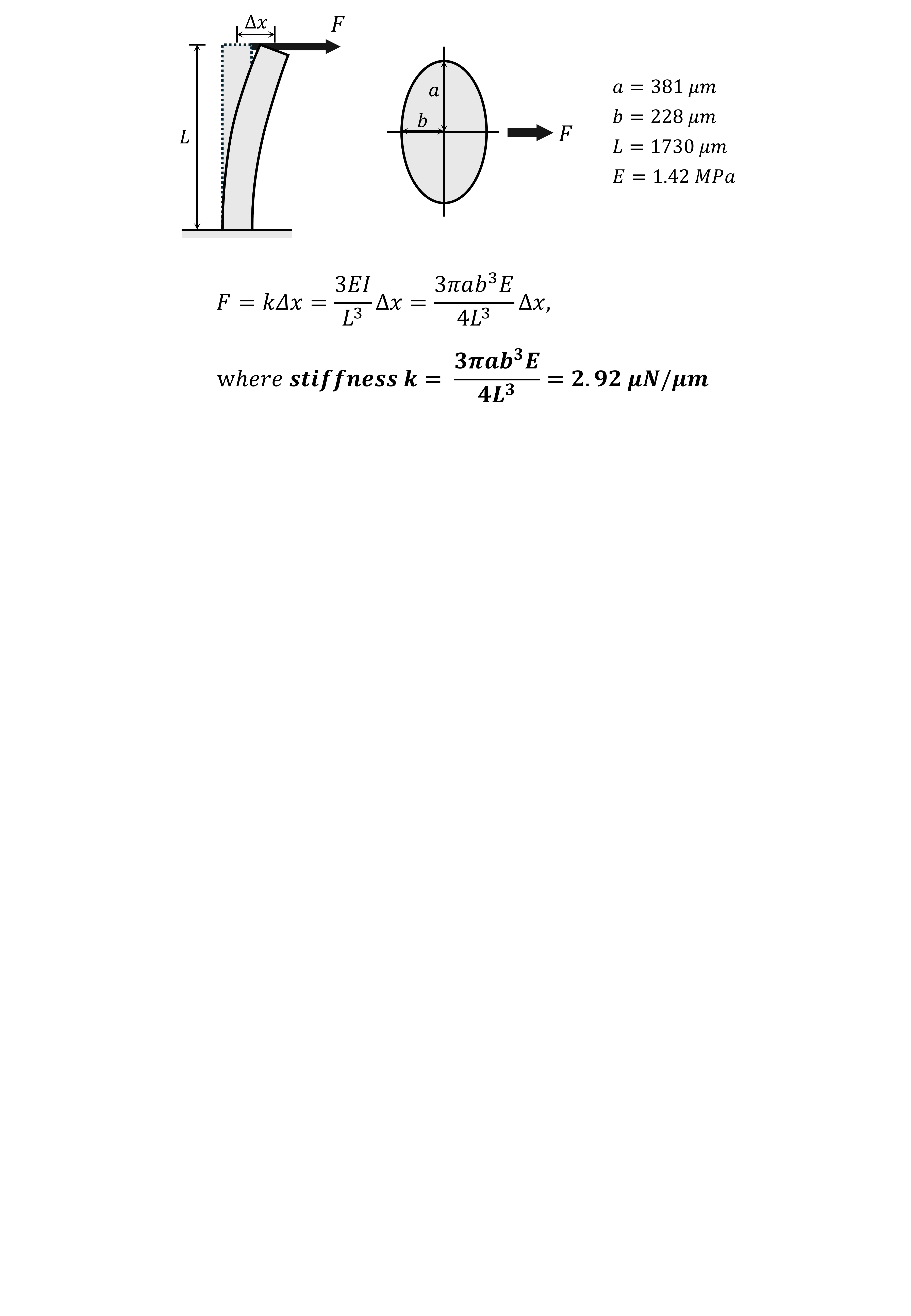


**Figure S8.** Analytical estimation of pillar stiffness. Stiffness was calculated assuming a concentrated force applied at the free end of the uncapped pillar, yielding 2.92 μN/μm.


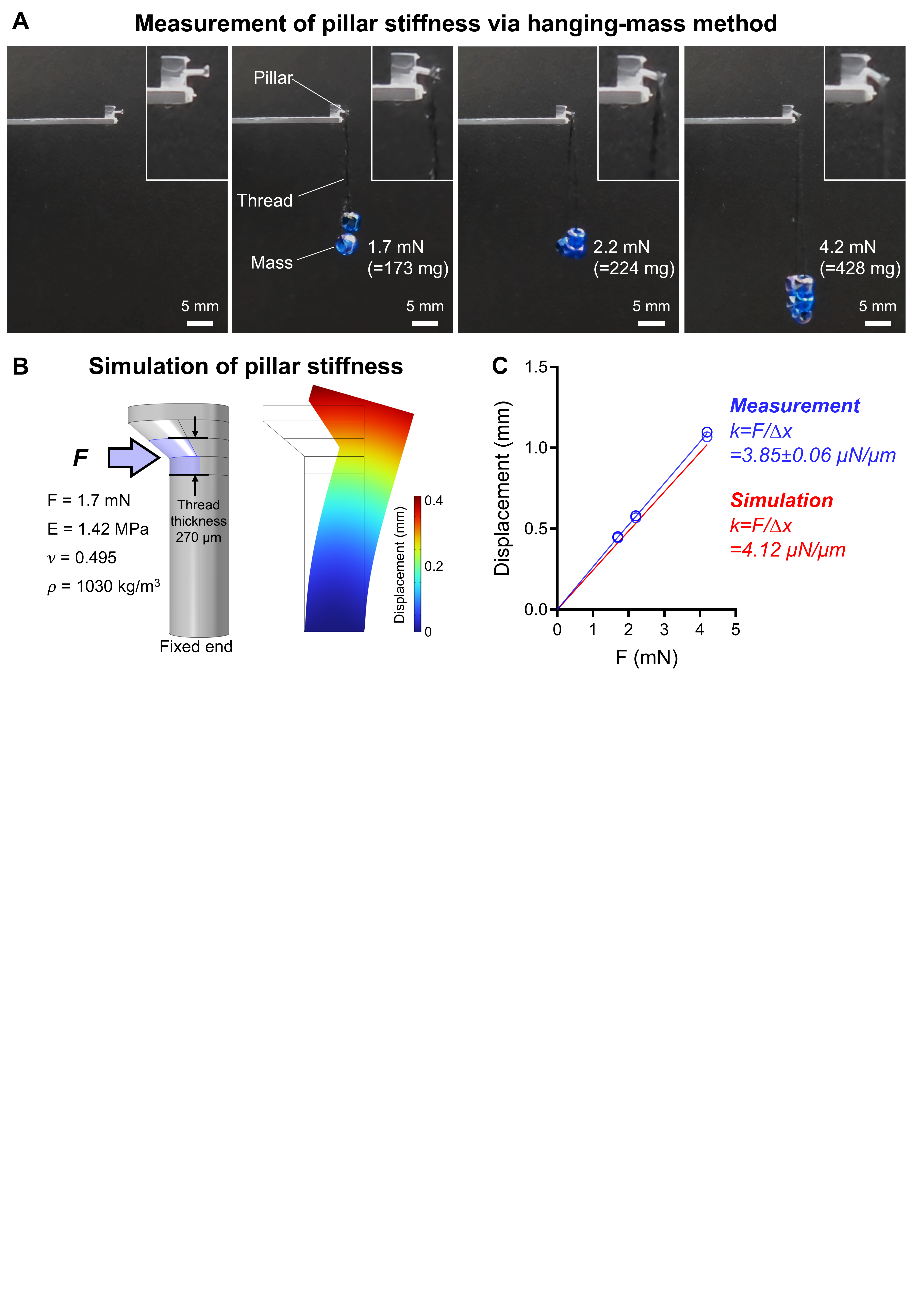


**Figure S9.** Comparison of measured and simulated pillar stiffness for validation of computational modeling. (A) Measurement of pillar stiffness using the hanging-mass method. Sequential images show a pillar loaded with plastic beads of known mass (0, 173, 224, and 428 mg), suspended from a thread looped beneath the cap to prevent slippage. Insets provide magnified views of the deflected pillars. Scale bars 5 mm. (B) Digital twin model replicating the experimental conditions in (A) for validation of the computational stiffness estimation. A force of 1.7 mN was applied at the thread–pillar contact surface, which had a thickness of 270 μm and was located beneath the cap. Details of the model are provided in *Materials and Methods*. (C) Force–displacement plots obtained from the measurements (n=3 independent samples) and simulation, shown in blue and red, respectively. The individual pillar stiffnesses were determined from the 1/slope of a linear regression constrained through the origin.


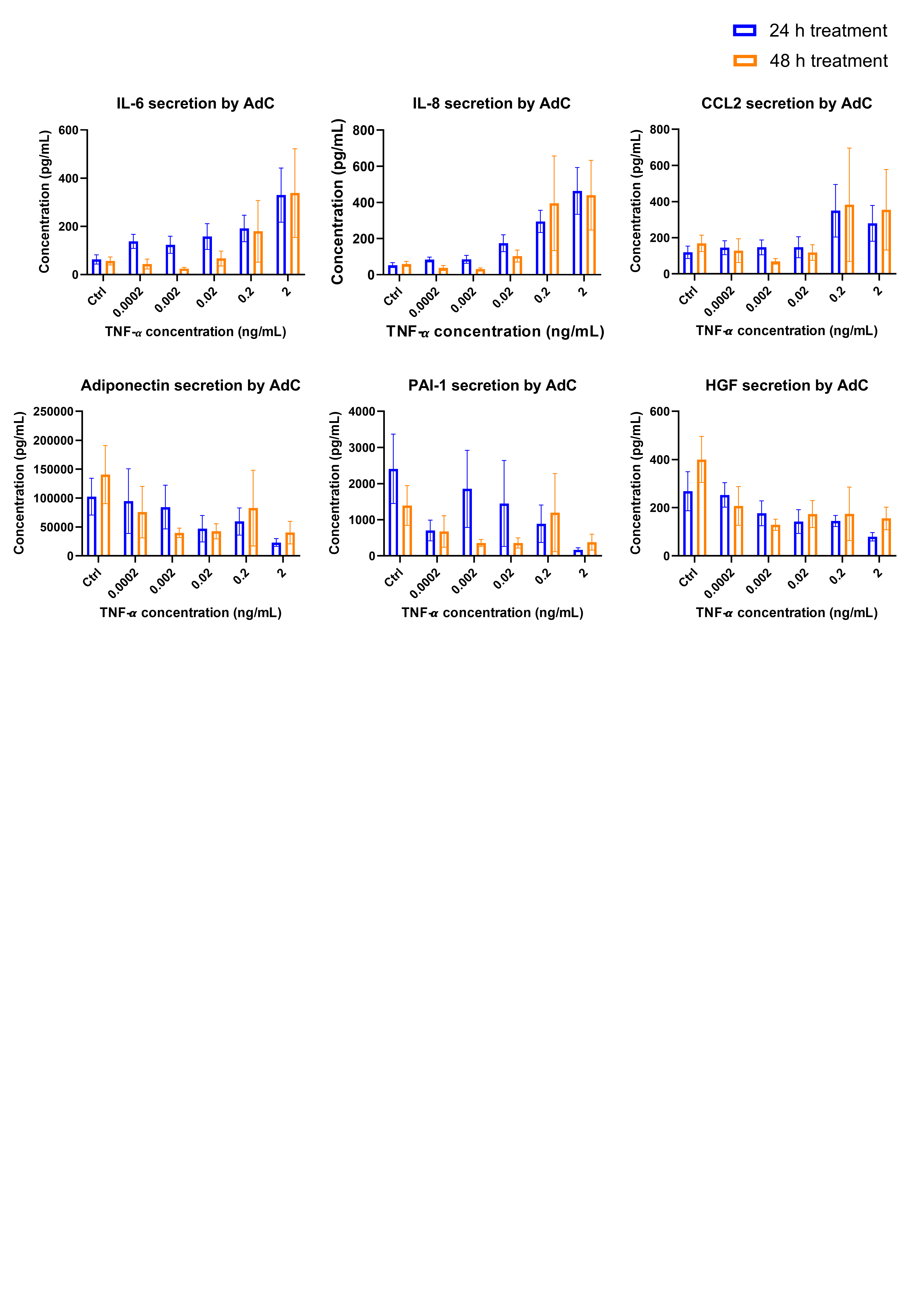


**Figure S10.** TNF-α induced adipocyte inflammation. Differentiated adipocytes were treated with serial dilutions of TNF-α for 24 h or 48 h. The secretion of adipokines (IL-6, IL-8, CCL2, adiponectin, PAI-1, and HGF) under different treatment conditions was quantified. n=4.


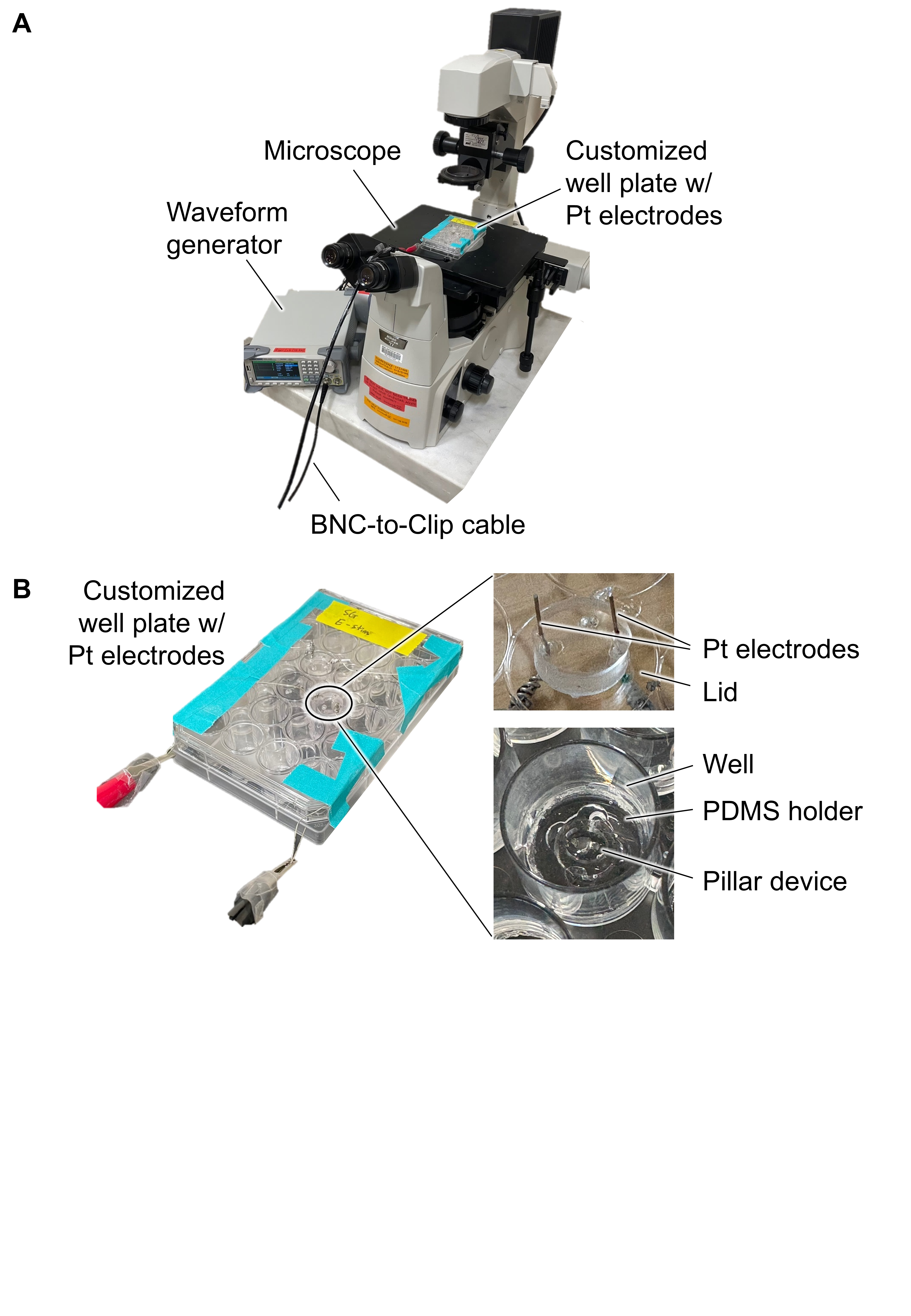


**Figure S11.** Customized E-Stim setup equipped with a microscope. (A) Overview of the entire setup and (B) its enlarged views of the lid and well of the 24-well plate.


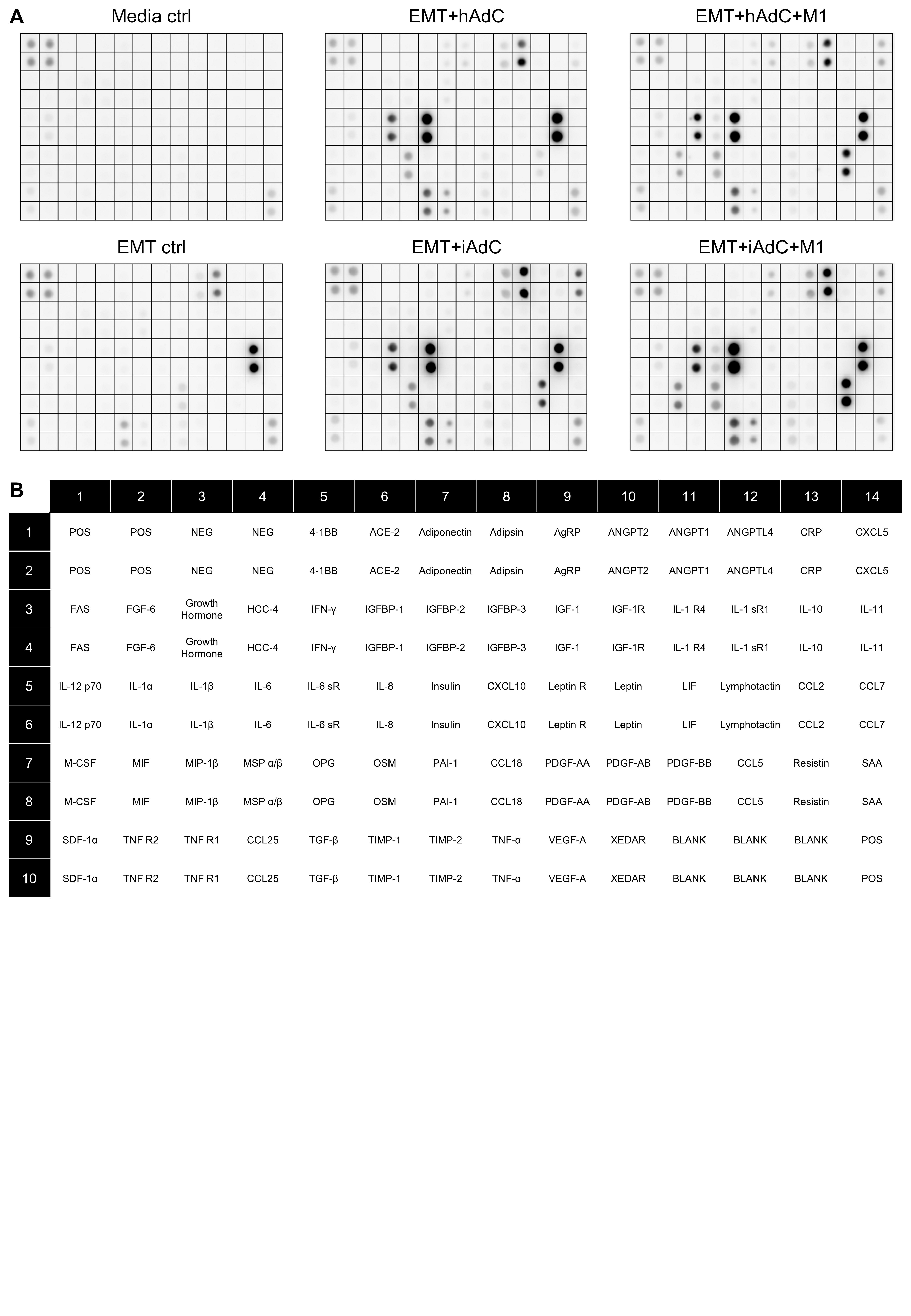


**Figure S12.** Human obesity cytokine array analysis for the detection of 62 adipokines in conditioned medium. (a) Representative array images. (b) Array spots corresponding to individual cytokines.


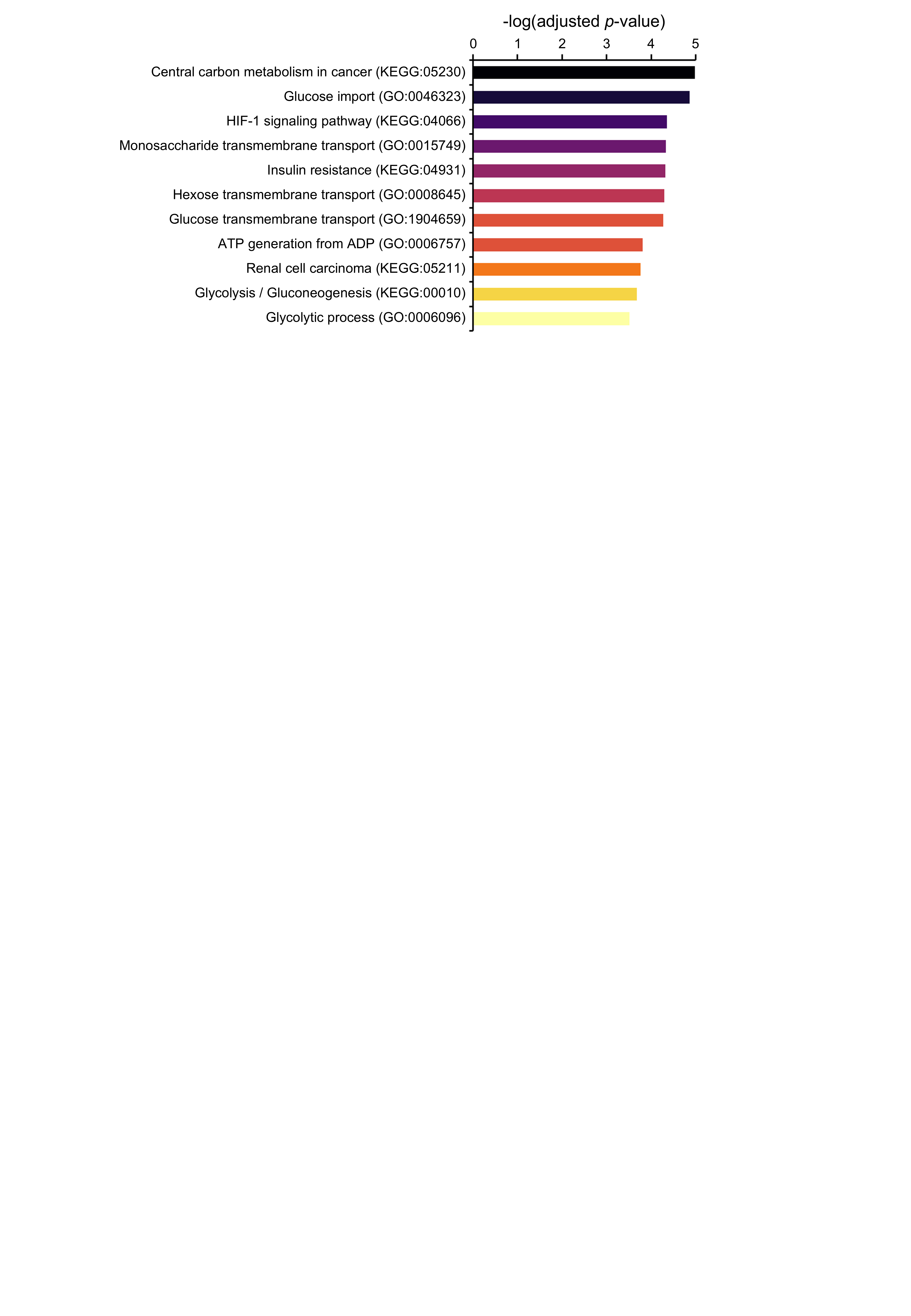


**Figure S13.** GO term and KEGG pathway enrichment analysis comparing the EMT–IAMC co-culture group with the EMT control group. The Y-axis displays the enriched GO terms and KEGG pathways, while the X-axis shows the negative base-10 logarithm of the Bonferroni-adjusted *p*-values, indicating statistical significance. Taller bars represent more significantly enriched terms.


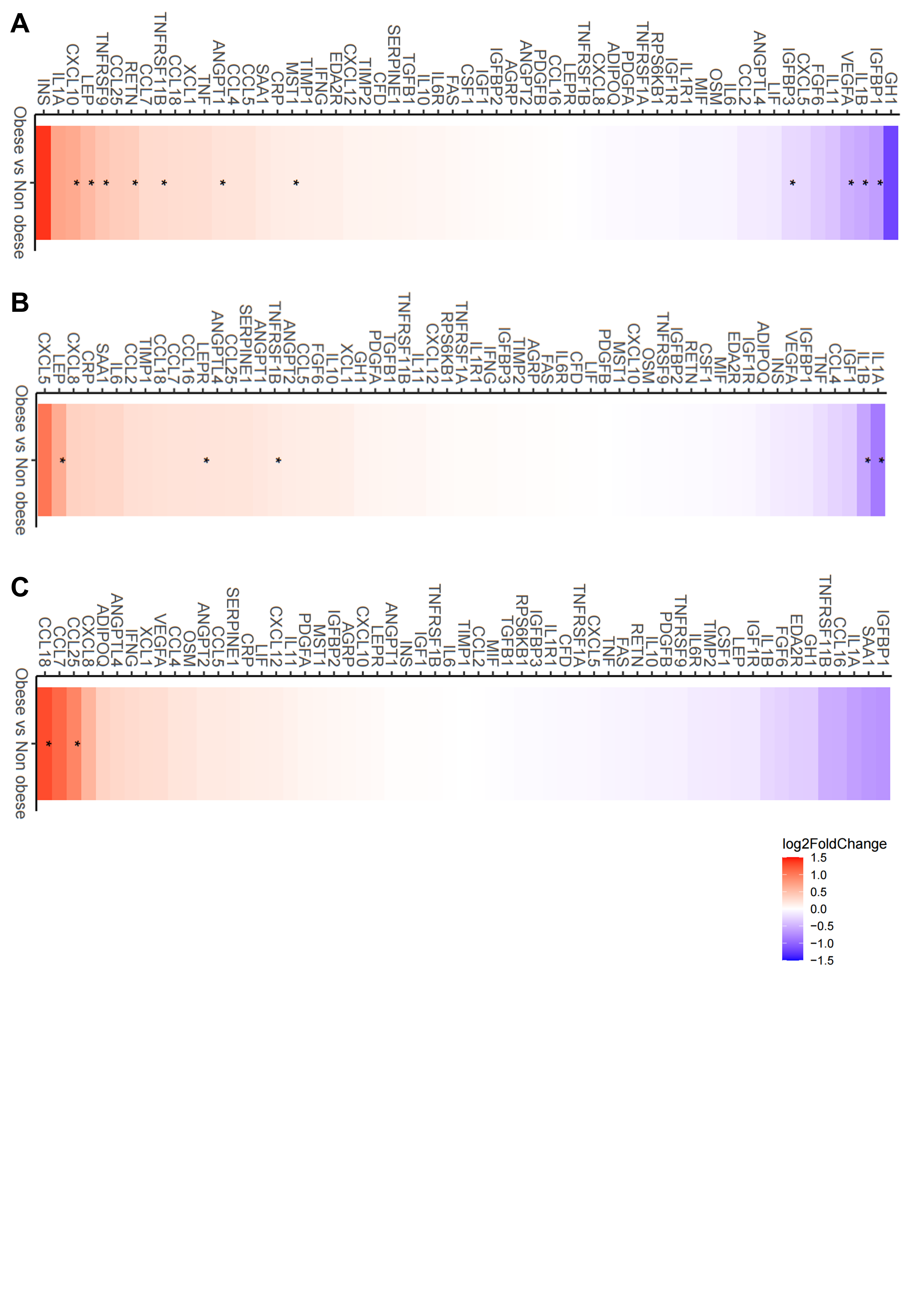


**Figure S14.** Heatmaps of selected DGEs comparing obese and non-obese patients in (A) subcutaneous adipose tissue, (B) visceral adipose tissue, and (C) whole blood. Genes with an adjusted *p*-value (FDR) < 0.05 were considered significantly differentially expressed.

**
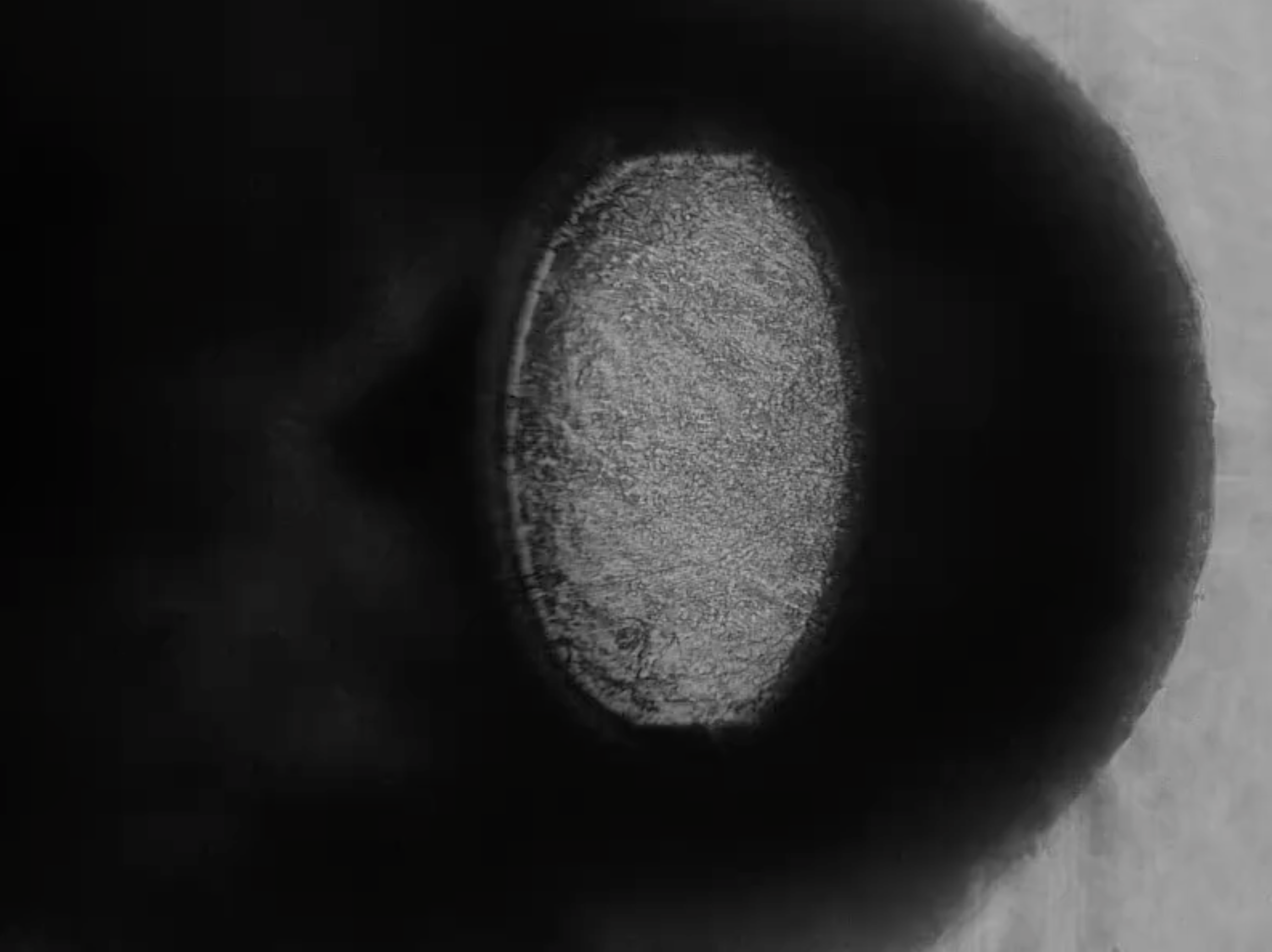
**

**Supporting Video 1.** Optical tracking of EMT contractile motion under E-Stim using a brightfield microscope with a 4× objective.

**
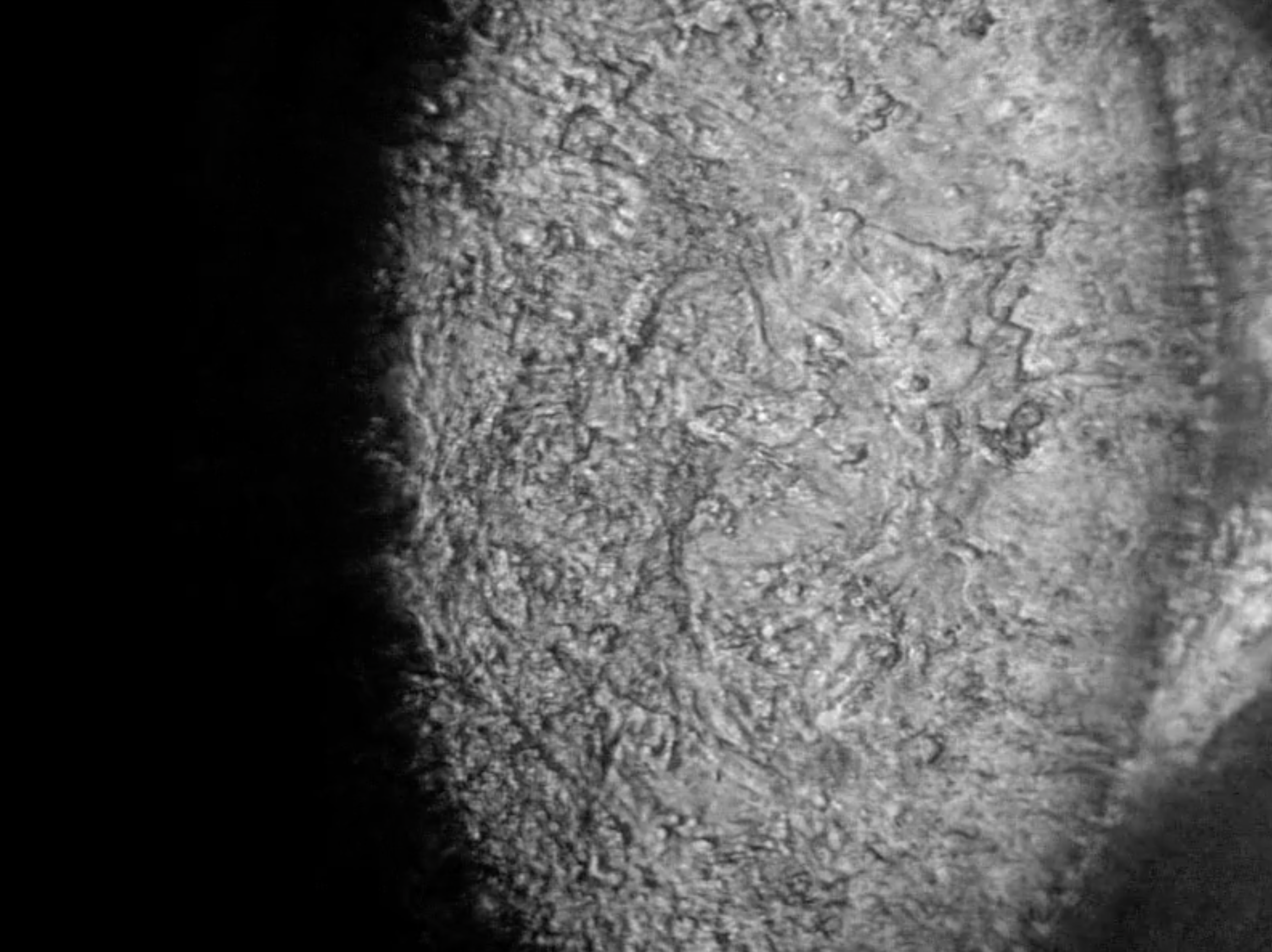
**

**Supporting Video 2.** Optical tracking of EMT contractile motion under E-Stim using a brightfield microscope with a 10× objective.
